# Supplementary material for: Rapid genomic surveillance of SARS-CoV-2 in a dense urban community of Kathmandu Valley using sewage samples
Source: PLoS One. 2023 Mar 30;18(3):e0283664. doi: 10.1371/journal.pone.0283664 (PMC10062583; doi:10.1371/journal.pone.0283664)
Supplement: S1 File — (DOCX) [file pone.0283664.s001.docx]

**S1 Table: Analysis of coronavirus detected in sewage samples**

| **Sample sites** | **Query ID** | **Accession** | **Organism** | **Evalue** | **Seqlen** | **Querycov** | **Identity** |
| --- | --- | --- | --- | --- | --- | --- | --- |
| TH1 | tig00000001 | [AL158839.11](https://www.ncbi.nlm.nih.gov/nucleotide/AL158839.11?report=genbank&log$=nucltop&blast_rank=1&RID=N1CNV44J01R) | Human | 1.00E-119 | 788 | 86 | 99.6 |
|  | tig00000002 | [KT254285.1](https://www.ncbi.nlm.nih.gov/nucleotide/KT254285.1?report=genbank&log$=nucltop&blast_rank=1&RID=N1CNV44J01R) | [Duck-dominant coronavirus](https://blast.ncbi.nlm.nih.gov/Blast.cgi#alnHdr_893675514) | 0 | 480 | 99 | 96.26 |
|  | tig00000003 | [KF293666.1](https://www.ncbi.nlm.nih.gov/nucleotide/KF293666.1?report=genbank&log$=nucltop&blast_rank=1&RID=N1CNV44J01R) | [Human coronavirus 229E](https://blast.ncbi.nlm.nih.gov/Blast.cgi#alnHdr_545899490) | 4.00E-78 | 466 | 44 | 92.96 |
|  | tig00000004 | [MT655131.1](https://www.ncbi.nlm.nih.gov/nucleotide/MT655131.1?report=genbank&log$=nucltop&blast_rank=1&RID=N1CNV44J01R) | SARS-CoV-2 | 0 | 472 | 92 | 98.17 |
|  | tig00000005 | [KF294370.1](https://www.ncbi.nlm.nih.gov/nucleotide/KF294370.1?report=genbank&log$=nucltop&blast_rank=1&RID=N1CNV44J01R) | Rat cov | 0 | 471 | 100 | 90.87 |
|  | tig00000006 | [MN306040.1](https://www.ncbi.nlm.nih.gov/nucleotide/MN306040.1?report=genbank&log$=nucltop&blast_rank=1&RID=N1CNV44J01R) | [Human coronavirus NL63](https://blast.ncbi.nlm.nih.gov/Blast.cgi#alnHdr_1726249351) | 0 | 391 | 100 | 99.49 |
|  | tig00000007 | no significant hits | |  |  |  |  |
| Te3 | tig00000001 | [LR824127.1](https://www.ncbi.nlm.nih.gov/nucleotide/LR824127.1?report=genbank&log$=nucltop&blast_rank=1&RID=N1HE2P7B014) | SARS-CoV-2 | 0 | 422 | 96 | 95.82 |
|  | tig00000001 | [KT254285.1](https://www.ncbi.nlm.nih.gov/nucleotide/KT254285.1?report=genbank&log$=nucltop&blast_rank=1&RID=N1CNV44J01R) | [Duck-dominant coronavirus](https://blast.ncbi.nlm.nih.gov/Blast.cgi#alnHdr_893675514) | 0 | 466 | 100 | 96.15 |
|  | tig00000002 | [KF294370.1](https://www.ncbi.nlm.nih.gov/nucleotide/KF294370.1?report=genbank&log$=nucltop&blast_rank=1&RID=N1CNV44J01R) | Rat cov | 2.00E-123 | 316 | 99 | 92.04 |
|  | tig00000003 | no significant hits | |  |  |  |  |
|  | tig00000004 | no significant hits | |  |  |  |  |
| TH2 | tig00000001 | [KT254285.1](https://www.ncbi.nlm.nih.gov/nucleotide/KT254285.1?report=genbank&log$=nucltop&blast_rank=1&RID=N1CNV44J01R) | [Duck-dominant coronavirus](https://blast.ncbi.nlm.nih.gov/Blast.cgi#alnHdr_893675514) | 0 | 475 | 99 | 96.2 |
|  | tig00000002 | [KF294370.1](https://www.ncbi.nlm.nih.gov/nucleotide/KF294370.1?report=genbank&log$=nucltop&blast_rank=1&RID=N1CNV44J01R) | Rat cov | 1.00E-125 | 327 | 99 | 91.38 |

**S2 Table: Estimated prevalence of SARS-CoV-2 per 1000 capita and population distribution of different sites.**

| S.N | Sampling Site | Pangolin lineage | population size | Sampling date | Viral load in Log10 | min incidence per 1000 | max incidence per 1000 | mean incidence per 1000 |
| --- | --- | --- | --- | --- | --- | --- | --- | --- |
| 1 | SA2 | B.1.36 | 81 | 18th Nov 2020 | 5.3639878 | 0.62 | 100.49 | 11.36 |
| 2 | SA7 | B.1.1 | 756 | 19th Nov 2020 | 4.797406 | 0.05 | 9.63 | 1.08 |
| 3 | CH4 | B.1.36 | 700 | 20th Nov 2020 | 4.4641914 | 0.06 | 9.69 | 1.1 |
| 4 | KU1 | B.1 | 1686 | 22nd Nov 2020 | 5.8682917 | 0.03 | 5.28 | 0.6 |
| 5 | SA6 | B.1.36 | 101 | 23rd Nov 2020 | 3.409595 | 0.3 | 51.29 | 5.74 |
| 6 | SA5 | B.1.1 | 400 | 24th Nov 2020 | 5.6841269 | 0.13 | 21.58 | 2.45 |
| 7 | SA4 | B.1.1 | 204 | 25th Nov 2020 | 4.3533391 | 0.2 | 32.4 | 3.68 |
| 8 | SA9 | B.1.1 | 227 | 27th Nov 2020 | 4.2814879 | 0.18 | 28.63 | 3.22 |
| 9 | GW11 | B.1.1 | 2367 | 9th Nov 2020 | 6.2869054 | 0.03 | 4.03 | 0.46 |
| 10 | GW12 | B.1.36 | - | 30th Nov 2020 | NA |  |  |  |
| 11 | GW14 | B.1.1 | 2000 | 11th Nov 2020 | 5.5375673 | 0.03 | 4.21 | 0.48 |
| 12 | GW15 | B.1 | 7000 | 1st Dec 2020 | 4.4665711 | 0.01 | 0.97 | 0.11 |
| 13 | GW13 | NA | 1500 | 10th sep 2020 | 4.2232363 | 0.03 | 4.27 | 0.48 |
| 14 | SA3 | NA | 191 | 26th nov 2020 | 4.1179338 | 0.21 | 32.72 | 3.72 |
| 15 | GW10 | NA | 532 | 27th Nov 2020 | 4.1931246 | 0.08 | 11.97 | 1.35 |
| 16 | SA1 | NA | 286 | 12th Nov 2020 | 3.372912 | 0.1 | 17.9 | 2.03 |

**S3 Table: SNP variant analysis of SARS-CoV-2, sequenced on MinION from CoV-Glue analysis (http://cov-glue.cvr.gla.ac.uk/). Frequency and percentage frequencies of nucleotides and deletions (-) were obtained using Geneious Prime**

| **Sample** | **Nucleotide mutation** | **Amino acid change** | **Gene** | **Frequency / Percentage of non-gaps (%)** | | | | | | | | **Frequency** | **% Frequency from total reads** |
| --- | --- | --- | --- | --- | --- | --- | --- | --- | --- | --- | --- | --- | --- |
|  |  |  |  | **A** | | **C** | | **G** | | **T** | | - | |
| SA2 | T1947C | V381A | NSP2 | 2 | 3.30% | 26 | 42.60% | 2 | 3.30% | 31 | 50.80% | 7 | 10.30% |
| SA2 | C18877T | L280L | Exonuclease | 1 | 2.60% | 2 | 5.30% | 1 | 2.60% | 34 | 89.50% | 2 | 5.00% |
| SA2 | G22992A | S477N | Spike | 26 | 81.30% | 0 | 0.00% | 3 | 9.40% | 3 | 9.40% | 7 | 17.90% |
| SA2 | G23270T | A570S | Spike | 6 | 11.30% | 3 | 5.70% | 11 | 20.80% | 33 | 62.30% | 5 | 8.60% |
| SA2 | A23403G | D614G | Spike | 6 | 17.10% | 2 | 5.70% | 25 | 71.40% | 2 | 5.70% | 9 | 20.50% |
| SA2 | G24781T | K1073N | Spike | 4 | 9.10% | 2 | 4.50% | 7 | 15.90% | 31 | 70.50% | 3 | 6.40% |
| SA2 | T25556G | V55G | ORF3a | 0 | 0.00% | 1 | 2.90% | 25 | 71.40% | 9 | 25.70% | 4 | 10.30% |
| SA2 | G25563T | Q57H | ORF3a | 3 | 8.10% | 0 | 0.00% | 2 | 5.40% | 32 | 86.50% | 2 | 5.10% |
| SA2 | C26735T | Y71Y | Membrane | 0 | 0.00% | 1 | 2.80% | 0 | 0.00% | 35 | 97.20% | 3 | 7.70% |
| SA2 | DEL: 17358-17359 | del_17358 | Helicase | 2 | 10.50% | 1 | 5.30% | 0 | 0.00% | 16 | 84.20% | 22 | 53.70% |
|  |  | del_17359 |  | 2 | 10.50% | 1 | 5.30% | 16 | 84.20% | 0 | 0.00% | 22 | 53.70% |
| SA7 | C5884T | Y1055Y | NSP3 | 0 | 0.00% | 2 | 9.50% | 0 | 0.00% | 19 | 90.50% | 1 | 4.50% |
| SA7 | G11230T | M86I | NSP6 | 0 | 0.00% | 0 | 0.00% | 1 | 2.70% | 36 | 97.30% | 2 | 5.10% |
| SA7 | G11609A | V213I | NSP6 | 15 | 55.60% | 0 | 0.00% | 12 | 44.40% | 0 | 0.00% | 2 | 6.90% |
| SA7 | A23403G | D614G | Spike | 7 | 18.90% | 0 | 0.00% | 27 | 73.00% | 3 | 8.10% | 7 | 15.90% |
| SA7 | G25621A | V77I | ORF3a | 32 | 82.10% | 1 | 2.60% | 6 | 15.40% | 0 | 0.00% | 0 | 0.00% |
| SA7 | C26060T | T223I | ORF3a | 0 | 0.00% | 8 | 22.20% | 1 | 2.80% | 27 | 75.00% | 3 | 7.70% |
| CH4 | C18877T | L280L | Exonuclease | 16 | 21.30% | 10 | 13.30% | 5 | 6.70% | 44 | 58.70% | 2 | 2.60% |
| CH4 | A23403G | D614G | Spike | 5 | 12.50% | 0 | 0.00% | 32 | 80.00% | 3 | 7.50% | 5 | 11.10% |
| CH4 | T25556G | V55G | ORF3a | 2 | 5.90% | 0 | 0.00% | 22 | 64.70% | 10 | 29.40% | 5 | 12.80% |
| CH4 | G25563T | Q57H | ORF3a | 1 | 2.80% | 0 | 0.00% | 3 | 8.30% | 32 | 88.90% | 3 | 7.70% |
| CH4 | C26060T | T223I | ORF3a | 1 | 2.90% | 2 | 5.70% | 0 | 0.00% | 32 | 91.40% | 4 | 10.30% |
| CH4 | C26192T | P267L | ORF3a | 0 | 0.00% | 4 | 10.80% | 1 | 2.70% | 32 | 86.50% | 2 | 5.10% |
| CH4 | C26735T | Y71Y | Membrane | 0 | 0.00% | 6 | 20.70% | 2 | 6.90% | 21 | 72.40% | 1 | 3.30% |
| KU1 | G922A | L39L | NSP2 | 44 | 59.50% | 11 | 14.90% | 15 | 20.30% | 4 | 5.40% | 2 | 2.60% |
| KU1 | G1782T | C326F | NSP2 | 0 | 0.00% | 1 | 3.30% | 2 | 6.70% | 27 | 90.00% | 9 | 23.10% |
| KU1 | T1947C | V381A | NSP2 | 2 | 2.70% | 27 | 37.00% | 0 | 0.00% | 44 | 60.30% | 4 | 5.20% |
| KU1 | A5608G | K963K | NSP3 | 16 | 57.10% | 0 | 0.00% | 11 | 39.30% | 1 | 3.60% | 2 | 6.70% |
| KU1 | C5884T | Y1055Y | NSP3 | 0 | 0.00% | 8 | 32.00% | 1 | 4.00% | 16 | 64.00% | 1 | 3.80% |
| KU1 | G11230T | M86I | NSP6 | 0 | 0.00% | 1 | 3.00% | 1 | 3.00% | 31 | 93.90% | 6 | 15.40% |
| KU1 | C18877T | L280L | Exonuclease | 2 | 5.00% | 3 | 7.50% | 0 | 0.00% | 35 | 87.50% | 2 | 4.80% |
| KU1 | A23403G | D614G | Spike | 5 | 13.90% | 3 | 8.30% | 24 | 66.70% | 4 | 11.10% | 11 | 23.40% |
| KU1 | C26060T | T223I | ORF3a | 0 | 0.00% | 1 | 5.60% | 1 | 5.60% | 16 | 88.90% | 8 | 30.80% |
| KU1 | DEL: 16965-16966 | del_16965 | NSP13 | 15 | 88.20% | 1 | 5.90% | 1 | 5.90% | 0 | 0.00% | 22 | 56.40% |
|  |  | del_16966 |  | 1 | 7.70% | 1 | 7.70% | 11 | 84.60% | 0 | 0.00% | 26 | 66.70% |
| KU1 | DEL: 25947 | del_25947 | ORF3a | 0 | 0.00% | 1 | 11.10% | 5 | 55.60% | 3 | 33.30% | 17 | 65.40% |
| SA5 | C5884T | Y1055Y | NSP3 | 0 | 0.00% | 5 | 12.50% | 1 | 2.50% | 34 | 85.00% | 0 | 0.00% |
| SA5 | G11230T | M86I | NSP6 | 0 | 0.00% | 1 | 2.90% | 2 | 5.90% | 31 | 91.20% | 5 | 12.80% |
| SA5 | G15380T | S647I | RdRp | 0 | 0.00% | 0 | 0.00% | 2 | 7.70% | 24 | 92.30% | 1 | 3.70% |
| SA5 | A23403G | D614G | Spike | 6 | 15.80% | 1 | 2.60% | 27 | 71.10% | 4 | 10.50% | 9 | 19.10% |
| SA5 | G25621A | V77I | ORF3a | 33 | 86.80% | 0 | 0.00% | 4 | 10.50% | 1 | 2.60% | 1 | 2.60% |
| SA5 | C26060T | T223I | ORF3a | 1 | 2.90% | 2 | 5.70% | 1 | 2.90% | 31 | 88.60% | 4 | 10.30% |
| SA5 | C29149T | I292I | Nucleocapsid | 0 | 0.00% | 1 | 2.90% | 0 | 0.00% | 33 | 97.10% | 5 | 12.80% |
| GW14 | G11230T | M86I | NSP6 | 1 | 2.80% | 0 | 0.00% | 12 | 33.30% | 23 | 63.90% | 3 | 7.70% |
| GW14 | A23403G | D614G | Spike | 7 | 18.40% | 2 | 5.30% | 25 | 65.80% | 4 | 10.50% | 14 | 26.90% |
| GW14 | G25621A | V77I | ORF3a | 32 | 82.10% | 0 | 0.00% | 6 | 15.40% | 1 | 2.60% | 1 | 2.50% |
| SA6 | T1947C | V381A | nsp2 | 2 | 4.50% | 22 | 50.00% | 2 | 4.50% | 18 | 40.90% | 12 | 21.40% |
| SA6 | G2782A | V21V | NSP3 | 33 | 94.30% | 0 | 0.00% | 2 | 5.70% | 0 | 0.00% | 4 | 10.30% |
| SA6 | G3431T | V238L | NSP3 | 0 | 0.00% | 0 | 0.00% | 4 | 11.10% | 32 | 88.90% | 3 | 7.70% |
| SA6 | C14408T | P323L | RdRp | 1 | 3.10% | 6 | 18.80% | 0 | 0.00% | 25 | 78.10% | 2 | 5.90% |
| SA6 | T15838A | W800R | NSP12 | 22 | 59.50% | 1 | 2.70% | 1 | 2.70% | 13 | 35.10% | 3 | 7.50% |
| SA6 | C18877T | L280L | Exonuclease | 0 | 0.00% | 3 | 8.30% | 0 | 0.00% | 33 | 91.70% | 3 | 7.70% |
| SA6 | A23403G | DG14G | Spike | 7 | 16.30% | 1 | 2.30% | 29 | 67.40% | 6 | 14.00% | 8 | 15.70% |
| SA6 | G25563T | Q57H | ORF3a | 3 | 8.10% | 1 | 2.70% | 4 | 10.80% | 29 | 78.40% | 3 | 7.50% |
| SA6 | C26735T | Y71Y | Membrane | 0 | 0.00% | 4 | 11.40% | 0 | 0.00% | 31 | 88.60% | 4 | 10.30% |
| SA6 | A28483G | Q70Q | Nucleocapsid | 33 | 50.80% | 6 | 9.20% | 25 | 38.50% | 1 | 1.50% | 0 | 0.00% |
| SA6 | DEL: codon 404 | del_2015 | NSP2 | 14 | 82.40% | 2 | 11.80% | 0 | 0.00% | 1 | 5.90% | 29 | 63.00% |
|  |  | del_2016 |  | 6 | 31.60% | 0 | 0 | 1 | 5.30% | 12 | 63.20% | 27 | 58.70% |
|  |  | del_2017 |  | 2 | 13.30% | 4 | 26.70% | 8 | 53.30% | 1 | 6.70% | 29 | 65.90% |
| SA6 | DEL: 24837 | del_24837 | Spike | 26 | 51.00% | 9 | 17.60% | 7 | 13.70% | 9 | 17.60% | 20 | 28.20% |
| SA4 | G984T | W60L | NSP2 | 4 | 6.30% | 0 | 0.00% | 24 | 37.50% | 36 | 56.30% | 13 | 16.90% |
| SA4 | G11230T | M86I | NSP6 | 0 | 0.00% | 1 | 3.10% | 1 | 3.10% | 30 | 93.80% | 7 | 17.90% |
| SA4 | C18877T | L280L | Exonuclease | 2 | 3.60% | 11 | 20.00% | 6 | 10.90% | 36 | 65.50% | 2 | 3.50% |
| SA4 | A23403G | D614G | Spike | 4 | 14.80% | 1 | 3.70% | 21 | 77.80% | 1 | 3.70% | 15 | 35.70% |
| SA4 | G28429T | W52C | N gene | 0 | 0.00% | 2 | 2.90% | 46 | 65.70% | 22 | 31.40% | 0 | 0.00% |
| SA9 | C8917T | F121F | NSP4 | 0 | 0.00% | 6 | 27.30% | 0 | 0.00% | 16 | 72.70% | 19 | 46.30% |
| SA9 | C14408T | P323L | RdRp | 0 | 0.00% | 7 | 18.40% | 0 | 0.00% | 31 | 81.60% | 1 | 2.60% |
| SA9 | C16111T | L891L | RdRp | 1 | 2.80% | 1 | 2.80% | 1 | 2.80% | 33 | 91.70% | 3 | 7.70% |
| SA9 | A23403G | D614G | Spike | 1 | 3.20% | 0 | 0.00% | 28 | 90.30% | 2 | 6.50% | 10 | 24.40% |
| SA9 | C26060T | T223I | ORF3a | 1 | 2.90% | 4 | 11.80% | 0 | 0.00% | 29 | 85.30% | 5 | 12.80% |
| SA9 | DEL: 9215 | del_9215 | NSP4 | 6 | 24.00% | 6 | 24.00% | 6 | 24.00% | 7 | 28.00% | 95 | 79.20% |
| GW12 | G922A | L39L | NSP2 | 29 | 40.80% | 23 | 32.40% | 6 | 8.50% | 13 | 18.30% | 2 | 2.70% |
| GW12 | T1947C | V381A | nsp2 | 4 | 5.60% | 26 | 36.10% | 2 | 2.80% | 40 | 55.60% | 5 | 6.50% |
| GW12 | C18877T | L280L | Exonuclease | 5 | 11.60% | 4 | 9.30% | 3 | 7.00% | 31 | 72.10% | 5 | 10.40% |
| GW12 | C21034T | L126F | O-ribose MTases | 0 | 0.00% | 7 | 23.30% | 0 | 0.00% | 23 | 76.70% | 7 | 23.30% |
| GW12 | A23403G | D614G | Spike | 4 | 13.30% | 2 | 6.70% | 21 | 70.00% | 3 | 10.00% | 12 | 28.60% |
| GW12 | G25563T | Q57H | ORF3a | 1 | 2.70% | 0 | 0.00% | 8 | 21.60% | 28 | 75.70% | 2 | 5.10% |
| GW12 | C26060T | T223I | ORF3a | 4 | 11.40% | 9 | 25.70% | 0 | 0.00% | 22 | 62.90% | 3 | 7.90% |
| GW12 | C26735T | Y71Y | Membrane | 1 | 2.70% | 2 | 5.40% | 1 | 2.70% | 33 | 89.20% | 2 | 5.10% |
| GW15 | G1274T | V157F | NSP2 | 0 | 0.00% | 0 | 0.00% | 3 | 10.00% | 27 | 90.00% | 26 | 46.40% |
| GW15 | C2695T | N630N | NSP2 | 0 | 0.00% | 5 | 13.50% | 0 | 0.00% | 32 | 86.50% | 2 | 5.10% |
| GW15 | C5884T | Y1055Y | NSP3 | 1 | 2.80% | 4 | 11.10% | 0 | 0.00% | 31 | 86.10% | 4 | 10.00% |
| GW15 | C6286T | T1189T | NSP3 | 0 | 0.00% | 12 | 33.30% | 1 | 2.80% | 23 | 63.90% | 4 | 10.00% |
| GW15 | G7936T | A1739A | NSP3 | 9 | 14.50% | 21 | 33.90% | 9 | 14.50% | 23 | 37.10% | 3 | 4.60% |
| GW15 | G11230T | M86I | NSP6 | 0 | 0.00% | 1 | 4.30% | 1 | 4.30% | 21 | 91.30% | 8 | 25.80% |
| GW15 | C11750T | L260F | NSP6 | 6 | 11.50% | 10 | 19.20% | 6 | 11.50% | 30 | 57.70% | 6 | 10.30% |
| GW15 | C14318T | T293I | RdRp | 12 | 16.00% | 16 | 21.30% | 2 | 2.70% | 45 | 60.00% | 1 | 1.30% |
| GW15 | C14408T | P323L | RdRp | 0 | 0.00% | 11 | 31.40% | 0 | 0.00% | 24 | 68.60% | 4 | 10.30% |
| GW15 | A23403G | D614G | Spike | 10 | 14.70% | 7 | 10.30% | 36 | 52.90% | 15 | 22.10% | 7 | 9.30% |
| GW15 | G25621A | V77I | ORF3a | 38 | 97.40% | 0 | 0.00% | 1 | 2.60% | 0 | 0.00% | 0 | 0.00% |
| GW15 | C26060T | T223I | ORF3a | 0 | 0.00% | 5 | 15.20% | 0 | 0.00% | 28 | 84.80% | 6 | 15.40% |
| GW11 | C835T | F10F | NSP2 | 0 | 0.00% | 10 | 43.50% | 0 | 0 | 13 | 56.50% | 16 | 41.00% |
| GW11 | C5884T | Y1055Y | NSP3 | 0 | 0.00% | 8 | 22.90% | 0 | 0 | 27 | 77.10% | 4 | 10.30% |
| GW11 | A23403G | D614G | Spike | 6 | 18.80% | 0 | 0 | 24 | 75% | 2 | 6.30% | 9 | 22.00% |

**S4 Table: Comparative data of 24 hour composite sampling and grab sampling.**

| **Sites** | **Grab** | |
| --- | --- | --- |
|  | **qPCR** | **Conventional** |
| Thapathali | Negative | Positive |
| Teku | Negative | Positive |
| Sankhamul | Negative | Positive |

**S5 Table: Detected SARS-CoV-2 mutations from TH1 and Te3 samples**

| Sample | Nucleotide.mutation | Mutation | Amino.acid.change | Frequency deteced | Gene | Type of mutation |
| --- | --- | --- | --- | --- | --- | --- |
| TH1 | T358c | 358: 't->c' | F31f | 2 | Nsp1 | Misense |
| TH1 | t418a |  | C51x |  | Nsp1 | Nonsense |
| TH1 | a436g |  | E57e |  | Nsp1 | Silent |
| TH1 | g2265a | 2265: 'g->a' | C487y | 7 | Nsp2 | Misense |
| TH1 | t3808c | 3808: 't->c' | N363n | 22 | Nsp3 | Silent |
| TH1 | a4038g |  | D1258g |  | Nsp3 | Misense |
| TH1 | a4131g |  | D1289g |  | Nsp3 | Misense |
| TH1 | t4240c | 4240: 't->c' | Y507y | 10 | Nsp3 | Silent |
| TH1 | t4323c |  | F1353s |  | Nsp3 | Misense |
| TH1 | a5606g | 5606: 'a->g' | K963e | 4 | Nsp3 | Misense |
| TH1 | a5898g |  | K1878r |  | Nsp3 | Misense |
| TH1 | a7496c | 7496: 'a->c' | M1593l | 3 | Nsp3 | Misense |
| TH1 | t7595c |  | F2444l |  | Nsp3 | Misense |
| TH1 | a8736g | 8736: 'a->g' | D61g | 1 | Nsp4 | Misense |
| TH1 | t8749c | 8749: 't->c' | A65a | 3 | Nsp4 | Silent |
| TH1 | a9829c |  | E3188d |  | 3cl | Misense |
| TH1 | t10397c | 10397: 't->c' | L115l | 2 | 3cl | Silent |
| TH1 | g10412t |  | G3383c |  | 3cl | Misense |
| TH1 | t10566c | 10566: 't->c' | V171a | 1 | 3cl | Misense |
| TH1 | a13341g | 13341: 'a->g' | D106g | 3 | Nsp10 | Misense |
| TH1 | t13737c | 13737: 't->c' | H99h | 71 | Rdrp | Silent |
| TH1 | t13750c |  | F4496l |  | Rdrp | Misense |
| TH1 | a13847g |  | E4528g |  | Rdrp | Misense |
| TH1 | t13952c | 13952: 't->c' | I171t | 8 | Rdrp | Misense |
| TH1 | a14240g | 14240: 'a->g' | K267r | 4 | Rdrp | Misense |
| TH1 | a14240t | 14240: 'a->t' | K267m | 3 | Rdrp | Misense |
| TH1 | t15563c |  | L5100s |  | Rdrp | Misense |
| TH1 | t17834c |  | V5857a |  | Helicase | Misense |
| TH1 | t17951c |  | I5896t |  | Helicase | Misense |
| TH1 | a23403g | 23403: 'a->g' | D614g | 192284 | Spike | Misense |
| TH1 | g24985a | 24985: 'g->a' | L1141l | 2 | Spike | Misense |
| TH1 | a25008g | 25008: 'a->g' | K1149r | 6 | Spike | Misense |
| TH1 | t29145c |  | L291p |  | Nucleocapsid | Misense |
| TH1 | a29147g | 29147: 'a->g' | I292v | 20 | Nucleocapsid | Misense |
| TH1 | a29221g | 29221: 'a->g' | G316g | 7 | Nucleocapsid | Silent |
| TH1 | g29262a |  | W330x |  | Nucleocapsid | Nonsense |
| TH1_minion | A23403g | 23403: 'a->g' | D614g | 192284 | Spike | Misense |
| Te3 | C2223t | 2223: 'c->t' | S473l | 11 | Nsp2 | Misense |
| Te3 | g2265a | 2265: 'g->a' | C487y | 7 | Nsp2 | Misense |
| Te3 | g3182a | 3182: 'g->a' | E155k | 11 | Nsp3 | Misense |
| Te3 | t4324g | 4324: 't->g' | F535l | 7 | Nsp3 | Misense |
| Te3 | a5606c |  | K1781q |  |  | Misense |
| Te3 | t5674c | 5674: 't->c' | P985p | 2 | Nsp3 | Silent |
| Te3 | c5842t | 5842: 'c->t' | Y1041y | 18 | Nsp3 | Silent |
| Te3 | c7471t | 7471: 'c->t' | D1584d | 55 | Nsp3 | Silent |
| Te3 | t8235c | 8235: 't->c' | L1839s | 1 | Nsp3 | Misense |
| Te3 | g8249a |  | D2662n |  | Nsp3 | Misense |
| Te3 | t10606c | 10606: 't->c' | P184p | 4 | 3cl | Silent |
| Te3 | a10973g | 10973: 'a->g' | S1g | 4 | Nsp6 | Misense |
| Te3 | a11282g |  | S3673g |  | Nsp6 | Misense |
| Te3 | a13263g |  | H4333r |  | Nsp10 | Misense |
| Te3 | a13555g |  | N4431d |  | Rdrp | Misense |
| Te3 | a13563c |  | K4433n |  | Rdrp | Misense |
| Te3 | a13563g | 13563: 'a->g' | K41k | 1 | Rdrp | Silent |
| Te3 | a13664c |  | H4467p |  | Rdrp | Misense |
| Te3 | c13968t | 13968: 'c->t' | A176a | 39 | Rdrp | Misense |
| Te3 | a14254g |  | K4664e |  | Rdrp | Misense |
| Te3 | t14628c |  | F4788f |  | Rdrp | Silent |
| Te3 | a14648g |  | N4795s |  | Rdrp | Misense |
| Te3 | a14774g | 14774: 'a->g' | D445g | 1 | Rdrp | Misense |
| Te3 | t18260a |  | I5999n |  | Exonuclease | Misense |
| Te3 | g18697a | 18697: 'g->a' | A220t | 2 | Exonuclease | Misense |
| Te3 | c18981t | 18981: 'c->t' | H314h | 238 | Exonuclease | Misense |
| Te3 | t19074c | 19074: 't->c' | D345d | 3 | Exonuclease | Misense |
| Te3 | g19231a | 19231: 'g->a' | V398i | 8 | Exonuclease | Misense |
| Te3 | t19258c |  | S6332p |  | Exonuclease | Misense |
| Te3 | g19272a | 19272: 'g->a' | L411l | 4 | Exonuclease | Misense |
| Te3 | a23403g | 23403: 'a->g' | D614g | 192284 | Spike | Misense |
| Te3 | g23517t | 23517: 'g->t' | G652v | 3 | Spike | Misense |
| Te3 | a24170g | 24170: 'a->g' | I870v | 88 | Spike | Misense |
| Te3 | t24391c |  | S943s |  | Spike | Misense |
| Te3 | t25084c | 25084: 't->c' | A1174a | 2 | Spike | Misense |
| Te3 | a25146g | 25146: 'a->g' | E1195g | 7 | Spike | Misense |
| Te3 | a25206g |  | Y1215c |  | Spike | Misense |
| Te3 | t25232c | 25232: 't->c' | L1224l | 6 | Spike | Misense |
| Te3 | a29155g |  | Q294q |  | Spike | Silent |
| Te3 | c29160t | 29160: 'c->t' | T296i | 39 | Nucleocapsid | Misense |
| Te3_minion | A23403g | 23403: 'a->g' | D614g | 192284 | Spike | Misense |

**S6 Table: Comparison of estimated and reported Covid-19 prevalence per 1000 capita in various sites in Lalitpur District from 27th November to 1st December 2020.**

| **Sampling Site** | **Date** | **Estimated prevalence per 1000 capita** | | | **Reported positive case in Lalitpur** | | | **Population of Lalitpur district** |
| --- | --- | --- | --- | --- | --- | --- | --- | --- |
|  |  | min prevalence per 1000 | max prevalence per 1000 | mean prevalence per 1000 | male | female | total | 468,132 |
| Gwarko |  |  |  |  |  |  |  | Prevalence rate per 1000 capita |
| GW10 | 27th Nov 2020 | 0.08 | 11.97 | 1.35 | 154 | 130 | 284 | 0.61 |
| GW11 | 9th Nov 2020 | 0.03 | 4.03 | 0.46 | 219 | 175 | 394 | 0.84 |
| GW13 | 10th Sep 2020 | 0.03 | 4.27 | 0.48 | 44 | 37 | 81 | 0.17 |
| GW14 | 11th Nov 2020 | 0.03 | 4.21 | 0.48 | 167 | 163 | 330 | 0.70 |
| GW15 | 1st Dec 2020 | 0.01 | 0.97 | 0.11 | 109 | 110 | 219 | 0.47 |
| ***Estimated Average prevalence per 1000 capita from 27th to 1st Dec 2020*** | | 0.036 | 5.09 | 0.576 | ***Reported Average prevalence per 1000 capita from 27th to 1st Dec 2020*** | | | 0.56 |
| Shankhamul |  |  |  |  |  |  |  |  |
| CH4 | 20th Nov 2020 | 0.06 | 9.69 | 1.1 | 174 | 165 | 339 | 0.72 |
| KU1 | 22nd Nov 2020 | 0.03 | 5.28 | 0.6 | 139 | 133 | 272 | 0.58 |
| SA1 | 12th Nov 2020 | 0.1 | 17.9 | 2.03 | 154 | 152 | 306 | 0.65 |
| SA2 | 18th Nov 2020 | 0.62 | 100.49 | 11.36 | 201 | 156 | 357 | 0.76 |
| SA3 | 26th Nov 2020 | 0.21 | 32.72 | 3.72 | 137 | 137 | 274 | 0.59 |
| SA4 | 25th Nov 2020 | 0.2 | 32.4 | 3.68 | 125 | 102 | 227 | 0.48 |
| SA5 | 24th Nov 2020 | 0.13 | 21.58 | 2.45 | 172 | 131 | 303 | 0.65 |
| SA6 | 23rd Nov 2020 | 0.3 | 51.29 | 5.74 | 181 | 150 | 331 | 0.71 |
| SA7 | 19th Nov 2020 | 0.05 | 9.63 | 1.08 | 195 | 168 | 363 | 0.78 |
| SA9 | 27th Nov 2020 | 0.18 | 28.63 | 3.22 | 154 | 130 | 284 | 0.61 |
| ***Estimated Average prevalence per 1000 capita from 27th to 1st Dec 2020*** | | 0.188 | 30.961 | 3.498 | ***Reported Average prevalence per 1000 capita from 20th to 27th November 2020*** | | | 0.65 |

**S7 Table: Sequencing depth and coverage data of the environmental samples sequenced on MinION and Illumina MiSeq.**

|  | **MinION Nanopore** | | | **Illumina MiSeq** | | |
| --- | --- | --- | --- | --- | --- | --- |
| **Sample name** | **Depth median (x)** | **% Coverage > 1x** | **% Coverage > 10x** | **Depth median (x)** | **% Coverage > 1x** | **% Coverage > 10x** |
| GW11 | 30 | 83.9 | 68.7 | 314 | 96 | 86 |
| GW12 | 14 | 76.7 | 55.3 | 183 | 96 | 84 |
| GW14 | 34 | 85.3 | 70.3 | 217 | 95 | 82 |
| GW15 | 36 | 92.9 | 86.2 | 900 | 100 | 99 |
| CH4 | 7 | 65.9 | 44.4 | NA | NA | NA |
| KU1 | 10 | 77.4 | 49.2 | 215 | 90 | 78 |
| SA2 | 5 | 65.6 | 39 | 126 | 84 | 71 |
| SA4 | 21 | 76.4 | 61 | 129 | 85 | 70 |
| SA5 | 16 | 81 | 57.9 | NA | NA | NA |
| SA6 | 10 | 70.2 | 49.6 | 1 | 51 | 29 |
| SA7 | 17 | 85.1 | 61.9 | 313 | 98 | 88 |
| SA9 | 3 | 58.5 | 34.7 | 9 | 70 | 49 |

**S8 Table: Pairwise comparison of Consensus sequence obtained from Miseq with MinION sequencer**

| Sample ID | Pairwise comparison score | Pairwise comparison % |
| --- | --- | --- |
| GW12 | 0.00000000 | 100.00 |
| GW14 | 0.00006424 | 100.00 |
| GW15 | 0.00000000 | 100.00 |
| KU1 | 0.00048717 | 100.00 |
| SA2 | 0.00000000 | 100.00 |
| SA4 | 0.00026427 | 100.00 |
| SA6 | 0.04238085 | 99.96 |
| SA7 | 0.00022894 | 100.00 |
| SA9 | 0.01436256 | 99.99 |
| GW11 | 0.00006500 | 100.00 |

**S9 Table: Detected mutations in SARS-CoV-2 samples from CoV-Glue analysis (http://cov-glue.cvr.gla.ac.uk/) using Illumina MiSeq. Frequency and percentage frequencies of nucleotides and deletions (-) were obtained using Geneious Prime and bam-readcount (https://github.com/genome/bam-readcount).**

| **Sample** | **Nucleotide mutation** | **Amino acid**  **substitution** | **Gene** | **Frequency / Percentage of non-gaps (%)** | | | | | | | | **Frequency** | **% Frequency from total reads** |
| --- | --- | --- | --- | --- | --- | --- | --- | --- | --- | --- | --- | --- | --- |
|  |  |  |  | **A** | | **C** | | **G** | | **T** | | - | |
| GW11 | T1947C | V381A | NSP2 | 1 | 0.07% | 432 | 31.65% | 2 | 0.15% | 930 | 68.13% | 0 | 0.00% |
| GW11 | C3037T | F106F | NSP3 | 0 | 0.00% | 0 | 0.00% | 0 | 0.00% | 11 | 100.00% | 0 | 0.00% |
| GW11 | A3069G | D117G | NSP3 | 10 | 71.43% | 0 | 0.00% | 4 | 28.57% | 0 | 0.00% | 0 | 0.00% |
| GW11 | G7798T | K1693N | NSP3 | 0 | 0.00% | 0 | 0.00% | 34 | 68.00% | 16 | 32.00% | 0 | 0.00% |
| GW11 | C14408T | P314L | NSP12b | 1 | 0.31% | 0 | 0.00% | 0 | 0.00% | 321 | 99.69% | 0 | 0.00% |
| GW11 | G15598A | V711I | NSP12b | 114 | 26.76% | 0 | 0.00% | 312 | 73.24% | 0 | 0.00% | 0 | 0.00% |
| GW11 | C18877T | C279C | NSP14 | 5 | 0.83% | 288 | 47.60% | 0 | 0.00% | 312 | 51.57% | 0 | 0.00% |
| GW11 | T21628A | T22T | Spike | 142 | 47.33% | 2 | 0.67% | 0 | 0.00% | 156 | 52.00% | 0 | 0.00% |
| GW11 | C21646T | Y28Y | Spike | 0 | 0.00% | 225 | 49.78% | 0 | 0.00% | 227 | 50.22% | 0 | 0.00% |
| GW11 | C22498T | I312I | Spike | 0 | 0.00% | 548 | 67.65% | 0 | 0.00% | 262 | 32.35% | 0 | 0.00% |
| GW11 | A23403G | D614G | Spike | 1 | 0.10% | 0 | 0.00% | 999 | 99.90% | 0 | 0.00% | 0 | 0.00% |
| GW11 | G25563T | Q57H | ORF3a | 0 | 0.00% | 0 | 0.00% | 265 | 62.35% | 160 | 37.65% | 0 | 0.00% |
| GW11 | G25621A | V77I | ORF3a | 79 | 26.60% | 0 | 0.00% | 218 | 73.40% | 0 | 0.00% | 0 | 0.00% |
| GW11 | C26060T | T223I | ORF3a | 0 | 0.00% | 492 | 46.77% | 0 | 0.00% | 560 | 53.23% | 0 | 0.00% |
| GW11 | C26735T | Y71Y | Membrane | 0 | 0.00% | 1285 | 72.03% | 0 | 0.00% | 499 | 27.97% | 0 | 0.00% |
| GW11 | A26896G | H125R | Membrane | 893 | 74.73% | 0 | 0.00% | 302 | 25.27% | 0 | 0.00% | 0 | 0.00% |
| GW11 | C28854T | S194L | Nucleocapsid | 0 | 0.00% | 1416 | 49.41% | 0 | 0.00% | 1450 | 50.59% | 0 | 0.00% |
| GW12 | G922A | L39L | NSP2 | 24 | 48.00% | 0 | 0.00% | 26 | 52.00% | 0 | 0.00% | 0 | 0.00% |
| GW12 | A1633C | K276N | NSP2 | 69 | 74.19% | 22 | 23.66% | 2 | 2.15% | 0 | 0.00% | 0 | 0.00% |
| GW12 | T1947C | V381A | NSP2 | 0 | 0.00% | 1726 | 87.97% | 1 | 0.05% | 235 | 11.98% | 0 | 0.00% |
| GW12 | G3431T | V238L | NSP3 | 0 | 0.00% | 0 | 0.00% | 32 | 44.44% | 40 | 55.56% | 0 | 0.00% |
| GW12 | C6402T | P1228L | NSP3 | 0 | 0.00% | 8 | 53.33% | 0 | 0.00% | 7 | 46.67% | 0 | 0.00% |
| GW12 | C7504T | Y1595Y | NSP3 | 0 | 0.00% | 175 | 66.54% | 0 | 0.00% | 88 | 33.46% | 0 | 0.00% |
| GW12 | T11015A | L15I | NSP6 | 12 | 35.29% | 0 | 0.00% | 0 | 0.00% | 22 | 64.71% | 0 | 0.00% |
| GW12 | C14408T | P314L | NSP12b | 0 | 0.00% | 1 | 0.64% | 0 | 0.00% | 155 | 99.36% | 0 | 0.00% |
| GW12 | C18877T | C279C | NSP14 | 0 | 0.00% | 15 | 7.58% | 0 | 0.00% | 183 | 92.42% | 0 | 0.00% |
| GW12 | C19220T | A394V | NSP14 | 4 | 0.34% | 447 | 37.98% | 0 | 0.00% | 726 | 61.68% | 0 | 0.00% |
| GW12 | T19516C | R492R | NSP14 | 0 | 0.00% | 37 | 26.24% | 0 | 0.00% | 104 | 73.76% | 0 | 0.00% |
| GW12 | T20746C | Y30H | NSP16 | 0 | 0.00% | 358 | 33.36% | 0 | 0.00% | 715 | 66.64% | 0 | 0.00% |
| GW12 | C21034T | L126F | NSP16 | 0 | 0.00% | 299 | 33.63% | 0 | 0.00% | 590 | 66.37% | 0 | 0.00% |
| GW12 | A21550G | N298D | NSP16 | 143 | 49.65% | 0 | 0.00% | 145 | 50.35% | 0 | 0.00% | 0 | 0.00% |
| GW12 | C21618G | T19R | Spike | 0 | 0.00% | 0 | 0.00% | 160 | 100.00% | 0 | 0.00% | 0 | 0.00% |
| GW12 | C21658T | F32F | Spike | 0 | 0.00% | 35 | 33.98% | 0 | 0.00% | 68 | 66.02% | 0 | 0.00% |
| GW12 | G21987A | G142D | Spike | 29 | 39.19% | 0 | 0.00% | 45 | 60.81% | 0 | 0.00% | 0 | 0.00% |
| GW12 | G22112A | G184S | Spike | 48 | 45.28% | 0 | 0.00% | 58 | 54.72% | 0 | 0.00% | 0 | 0.00% |
| GW12 | C22444T | D294D | Spike | 0 | 0.00% | 35 | 14.96% | 0 | 0.00% | 199 | 85.04% | 0 | 0.00% |
| GW12 | A23403G | D614G | Spike | 0 | 0.00% | 0 | 0.00% | 150 | 100.00% | 0 | 0.00% | 0 | 0.00% |
| GW12 | C23604G | P681R | Spike | 0 | 0.00% | 388 | 59.78% | 261 | 40.22% | 0 | 0.00% | 0 | 0.00% |
| GW12 | T25556G | V55G | ORF3a | 0 | 0.00% | 0 | 0.00% | 33 | 27.97% | 85 | 72.03% | 0 | 0.00% |
| GW12 | G25563T | Q57H | ORF3a | 0 | 0.00% | 0 | 0.00% | 0 | 0.00% | 114 | 100.00% | 0 | 0.00% |
| GW12 | C26060T | T223I | ORF3a | 0 | 0.00% | 446 | 33.26% | 0 | 0.00% | 895 | 66.74% | 0 | 0.00% |
| GW12 | C26735T | Y71Y | Membrane | 5 | 0.15% | 1095 | 33.59% | 3 | 0.09% | 2157 | 66.17% | 0 | 0.00% |
| GW12 | A27373T | M58L | ORF6 | 2276 | 61.40% | 1 | 0.03% | 7 | 0.19% | 1423 | 38.39% | 0 | 0.00% |
| GW12 | C28854T | S194L | Nucleocapsid | 0 | 0.00% | 466 | 36.75% | 0 | 0.00% | 802 | 63.25% | 0 | 0.00% |
| GW12 | T29564C | Y3H | ORF10 | 0 | 0.00% | 11 | 36.67% | 0 | 0.00% | 19 | 63.33% | 0 | 0.00% |
| GW12 | DEL: 29594-29595 | del_29594 | ORF10 | 3 | 100.00% | 0 | 0.00% | 0 | 0.00% | 0 | 0.00% | 29 | 90.60% |
| GW12 |  | del_29595 |  | 0 | 0.00% | 0 | 0.00% | 0 | 0.00% | 3 | 100.00% | 29 | 90.60% |
| GW14 | C745T | N160N | NSP1 | 0 | 0.00% | 100 | 40.82% | 0 | 0.00% | 145 | 59.18% | 0 | 0.00% |
| GW14 | G1274T | V157F | NSP2 | 1 | 0.13% | 0 | 0.00% | 541 | 70.81% | 222 | 29.06% | 0 | 0.00% |
| GW14 | A1328G | T175A | NSP2 | 245 | 56.58% | 0 | 0.00% | 187 | 43.19% | 1 | 0.23% | 0 | 0.00% |
| GW14 | T1344C | L180S | NSP2 | 0 | 0.00% | 14 | 63.64% | 0 | 0.00% | 8 | 36.36% | 0 | 0.00% |
| GW14 | T3143A | L142M | NSP3 | 37 | 27.61% | 3 | 2.24% | 0 | 0.00% | 94 | 70.15% | 0 | 0.00% |
| GW14 | A4228T | P503P | NSP3 | 32 | 66.67% | 0 | 0.00% | 0 | 0.00% | 16 | 33.33% | 0 | 0.00% |
| GW14 | C7124T | P1469S | NSP3 | 0 | 0.00% | 116 | 46.96% | 0 | 0.00% | 131 | 53.04% | 0 | 0.00% |
| GW14 | T7183C | T1488T | NSP3 | 0 | 0.00% | 109 | 27.18% | 0 | 0.00% | 292 | 72.82% | 0 | 0.00% |
| GW14 | C8986T | D144D | NSP4 | 0 | 0.00% | 87 | 72.50% | 0 | 0.00% | 33 | 27.50% | 0 | 0.00% |
| GW14 | G9053T | V167L | NSP4 | 0 | 0.00% | 0 | 0.00% | 122 | 68.16% | 57 | 31.84% | 0 | 0.00% |
| GW14 | A10127G | T25A | NSP5 | 132 | 61.97% | 0 | 0.00% | 81 | 38.03% | 0 | 0.00% | 0 | 0.00% |
| GW14 | G10266T | G71V | NSP5 | 2 | 0.37% | 0 | 0.00% | 344 | 64.42% | 188 | 35.21% | 0 | 0.00% |
| GW14 | A11201G | T77A | NSP6 | 586 | 29.76% | 0 | 0.00% | 1382 | 70.19% | 1 | 0.05% | 0 | 0.00% |
| GW14 | A11332G | V120V | NSP6 | 420 | 26.58% | 1 | 0.06% | 1159 | 73.35% | 0 | 0.00% | 0 | 0.00% |
| GW14 | G12802A | R39R | NSP9 | 143 | 41.81% | 0 | 0.00% | 199 | 58.19% | 0 | 0.00% | 0 | 0.00% |
| GW14 | C14262T | D265D | NSP12b | 0 | 0.00% | 90 | 60.00% | 0 | 0.00% | 60 | 40.00% | 0 | 0.00% |
| GW14 | C14408T | P314L | NSP12b | 0 | 0.00% | 0 | 0.00% | 0 | 0.00% | 339 | 100.00% | 0 | 0.00% |
| GW14 | A14748G | E427E | NSP12b | 94 | 72.87% | 0 | 0.00% | 35 | 27.13% | 0 | 0.00% | 0 | 0.00% |
| GW14 | A14943C | S492S | NSP12b | 97 | 74.05% | 34 | 25.95% | 0 | 0.00% | 0 | 0.00% | 0 | 0.00% |
| GW14 | C16466T | P77L | NSP13 | 0 | 0.00% | 358 | 64.86% | 0 | 0.00% | 194 | 35.14% | 0 | 0.00% |
| GW14 | C18877T | C279C | NSP14 | 0 | 0.00% | 298 | 36.70% | 0 | 0.00% | 514 | 63.30% | 0 | 0.00% |
| GW14 | C21618G | T19R | Spike | 0 | 0.00% | 197 | 39.24% | 305 | 60.76% | 0 | 0.00% | 0 | 0.00% |
| GW14 | C21646T | Y28Y | Spike | 18 | 4.62% | 270 | 69.23% | 0 | 0.00% | 102 | 26.15% | 0 | 0.00% |
| GW14 | G21987A | G142D | Spike | 22 | 50.00% | 0 | 0.00% | 22 | 50.00% | 0 | 0.00% | 0 | 0.00% |
| GW14 | C22444T | D294D | Spike | 0 | 0.00% | 90 | 55.90% | 0 | 0.00% | 71 | 44.10% | 0 | 0.00% |
| GW14 | A23403G | D614G | Spike | 0 | 0.00% | 0 | 0.00% | 51 | 100.00% | 0 | 0.00% | 0 | 0.00% |
| GW14 | C23604G | P681R | Spike | 0 | 0.00% | 107 | 27.02% | 289 | 72.98% | 0 | 0.00% | 0 | 0.00% |
| GW14 | C25452T | I20I | ORF3a | 0 | 0.00% | 139 | 71.65% | 0 | 0.00% | 55 | 28.35% | 0 | 0.00% |
| GW14 | C25469T | S26L | ORF3a | 1 | 0.39% | 117 | 45.70% | 0 | 0.00% | 138 | 53.91% | 0 | 0.00% |
| GW14 | G25621A | V77I | ORF3a | 58 | 27.62% | 0 | 0.00% | 152 | 72.38% | 0 | 0.00% | 0 | 0.00% |
| GW14 | C26060T | T223I | ORF3a | 0 | 0.00% | 367 | 55.11% | 0 | 0.00% | 299 | 44.89% | 0 | 0.00% |
| GW14 | T26767C | I82T | Membrane | 0 | 0.00% | 732 | 35.36% | 0 | 0.00% | 1338 | 64.64% | 0 | 0.00% |
| GW14 | A27373T | M58L | ORF6 | 858 | 38.32% | 1 | 0.04% | 2 | 0.09% | 1378 | 61.55% | 0 | 0.00% |
| GW14 | C27389T | 27389 | 3'UTR | 3 | 0.15% | 1360 | 68.10% | 0 | 0.00% | 634 | 31.75% | 0 | 0.00% |
| GW14 | C27752T | T120I | ORF7a | 0 | 0.00% | 55 | 43.31% | 0 | 0.00% | 72 | 56.69% | 0 | 0.00% |
| GW14 | C27874T | T40I | ORF7b | 0 | 0.00% | 70 | 29.05% | 0 | 0.00% | 171 | 70.95% | 0 | 0.00% |
| GW14 | A28413G | N47S | Nucleocapsid | 370 | 71.57% | 0 | 0.00% | 147 | 28.43% | 0 | 0.00% | 0 | 0.00% |
| GW14 | T28424C | S51P | Nucleocapsid | 0 | 0.00% | 122 | 29.33% | 0 | 0.00% | 294 | 70.67% | 0 | 0.00% |
| GW14 | A28461G | D63G | Nucleocapsid | 180 | 45.92% | 0 | 0.00% | 212 | 54.08% | 0 | 0.00% | 0 | 0.00% |
| GW14 | C28854T | S194L | Nucleocapsid | 0 | 0.00% | 1304 | 48.44% | 1 | 0.04% | 1387 | 51.52% | 0 | 0.00% |
| GW14 | G29402T | D377Y | Nucleocapsid | 1 | 0.09% | 0 | 0.00% | 732 | 67.97% | 344 | 31.94% | 0 | 0.00% |
| GW14 | T29626C | S23S | ORF10 | 0 | 0.00% | 190 | 31.20% | 1 | 0.16% | 418 | 68.64% | 0 | 0.00% |
| GW15 | G1274T | V157F | NSP2 | 0 | 0.00% | 1 | 0.25% | 1 | 0.25% | 397 | 99.50% | 0 | 0.00% |
| GW15 | C2695T | N630N | NSP2 | 0 | 0.00% | 0 | 0.00% | 0 | 0.00% | 359 | 100.00% | 0 | 0.00% |
| GW15 | C3037T | F106F | NSP3 | 0 | 0.00% | 0 | 0.00% | 0 | 0.00% | 172 | 100.00% | 0 | 0.00% |
| GW15 | C5884T | Y1055Y | NSP3 | 0 | 0.00% | 8 | 9.20% | 0 | 0.00% | 79 | 90.80% | 0 | 0.00% |
| GW15 | C6286T | T1189T | NSP3 | 0 | 0.00% | 1 | 1.96% | 0 | 0.00% | 50 | 98.04% | 0 | 0.00% |
| GW15 | G7936T | A1739A | NSP3 | 0 | 0.00% | 0 | 0.00% | 1 | 0.19% | 525 | 99.81% | 0 | 0.00% |
| GW15 | G11230T | M86I | NSP6 | 1 | 0.03% | 9 | 0.28% | 20 | 0.61% | 3226 | 99.08% | 0 | 0.00% |
| GW15 | C11750T | L260F | NSP6 | 2 | 0.14% | 20 | 1.45% | 0 | 0.00% | 1361 | 98.41% | 0 | 0.00% |
| GW15 | C14318T | T284I | NSP12b | 0 | 0.00% | 1 | 0.07% | 1 | 0.07% | 1372 | 99.85% | 0 | 0.00% |
| GW15 | C14408T | P314L | NSP12b | 0 | 0.00% | 5 | 0.56% | 0 | 0.00% | 882 | 99.44% | 0 | 0.00% |
| GW15 | T19516C | R492R | NSP14 | 1 | 0.13% | 187 | 23.79% | 1 | 0.13% | 597 | 75.95% | 0 | 0.00% |
| GW15 | T21285C | C209C | NSP16 | 0 | 0.00% | 128 | 100.00% | 0 | 0.00% | 0 | 0.00% | 0 | 0.00% |
| GW15 | C21646T | Y28Y | Spike | 0 | 0.00% | 91 | 9.25% | 0 | 0.00% | 893 | 90.75% | 0 | 0.00% |
| GW15 | A23403G | D614G | Spike | 4 | 0.31% | 0 | 0.00% | 1279 | 99.69% | 0 | 0.00% | 0 | 0.00% |
| GW15 | G25621A | V77I | ORF3a | 1377 | 99.85% | 0 | 0.00% | 2 | 0.15% | 0 | 0.00% | 0 | 0.00% |
| GW15 | C25667T | S92L | ORF3a | 0 | 0.00% | 314 | 37.65% | 0 | 0.00% | 520 | 62.35% | 0 | 0.00% |
| GW15 | C26060T | T223I | ORF3a | 0 | 0.00% | 26 | 1.68% | 1 | 0.06% | 1518 | 98.25% | 0 | 0.00% |
| GW15 | G28881A | R203K | Nucleocapsid | 121 | 74.23% | 0 | 0.00% | 7 | 4.29% | 35 | 21.47% | 0 | 0.00% |
| GW15 | G28881T | RG203IR | Nucleocapsid | 121 | 74.23% | 0 | 0.00% | 7 | 4.29% | 35 | 21.47% | 0 | 0.00% |
| GW15 | G28882A |  |  | 115 | 73.72% | 0 | 0.00% | 41 | 26.28% | 0 | 0.00% | 0 | 0.00% |
| GW15 | G28883C |  |  | 0 | 0.00% | 121 | 77.56% | 35 | 22.44% | 0 | 0.00% | 0 | 0.00% |
| KU1 | C745T | N160N | NSP1 | 0 | 0.00% | 219 | 65.77% | 0 | 0.00% | 114 | 34.23% | 0 | 0.00% |
| KU1 | G922A | L39L | NSP2 | 177 | 25.95% | 0 | 0.00% | 505 | 74.05% | 0 | 0.00% | 0 | 0.00% |
| KU1 | A1691G | I296V | NSP2 | 95 | 36.96% | 0 | 0.00% | 162 | 63.04% | 0 | 0.00% | 0 | 0.00% |
| KU1 | A1799G | T332A | NSP2 | 85 | 43.37% | 1 | 0.51% | 110 | 56.12% | 0 | 0.00% | 0 | 0.00% |
| KU1 | C3037T | F106F | NSP3 | 0 | 0.00% | 0 | 0.00% | 0 | 0.00% | 12 | 100.00% | 0 | 0.00% |
| KU1 | A3075G | E119G | NSP3 | 10 | 62.50% | 0 | 0.00% | 6 | 37.50% | 0 | 0.00% | 0 | 0.00% |
| KU1 | A3387C | K223T | NSP3 | 105 | 62.50% | 62 | 36.90% | 1 | 0.60% | 0 | 0.00% | 0 | 0.00% |
| KU1 | T4660C | Y647Y | NSP3 | 0 | 0.00% | 48 | 34.04% | 0 | 0.00% | 93 | 65.96% | 0 | 0.00% |
| KU1 | A4864C | K715N | NSP3 | 287 | 64.79% | 151 | 34.09% | 4 | 0.90% | 1 | 0.23% | 0 | 0.00% |
| KU1 | G5180A | D821N | NSP3 | 64 | 33.33% | 0 | 0.00% | 128 | 66.67% | 0 | 0.00% | 0 | 0.00% |
| KU1 | C7504T | Y1595Y | NSP3 | 0 | 0.00% | 22 | 55.00% | 0 | 0.00% | 18 | 45.00% | 0 | 0.00% |
| KU1 | T7813C | H1698H | NSP3 | 0 | 0.00% | 16 | 34.78% | 0 | 0.00% | 30 | 65.22% | 0 | 0.00% |
| KU1 | C8986T | D144D | NSP4 | 0 | 0.00% | 24 | 6.74% | 1 | 0.28% | 331 | 92.98% | 0 | 0.00% |
| KU1 | G9053T | V167L | NSP4 | 0 | 0.00% | 4 | 0.55% | 40 | 5.47% | 687 | 93.98% | 0 | 0.00% |
| KU1 | T10749C | L232P | NSP5 | 0 | 0.00% | 23 | 25.27% | 0 | 0.00% | 68 | 74.73% | 0 | 0.00% |
| KU1 | T12037C | A65A | NSP7 | 0 | 0.00% | 125 | 36.66% | 0 | 0.00% | 216 | 63.34% | 0 | 0.00% |
| KU1 | C14408T | P314L | NSP12b | 1 | 0.26% | 0 | 0.00% | 0 | 0.00% | 379 | 99.74% | 0 | 0.00% |
| KU1 | A15887G | H807R | NSP12b | 1088 | 61.50% | 1 | 0.06% | 679 | 38.38% | 1 | 0.06% | 0 | 0.00% |
| KU1 | G16243A | G3R | NSP13 | 124 | 24.17% | 0 | 0.00% | 389 | 75.83% | 0 | 0.00% | 0 | 0.00% |
| KU1 | C16466T | P77L | NSP13 | 0 | 0.00% | 182 | 65.00% | 0 | 0.00% | 98 | 35.00% | 0 | 0.00% |
| KU1 | T16542C | N102N | NSP13 | 0 | 0.00% | 246 | 56.04% | 0 | 0.00% | 193 | 43.96% | 0 | 0.00% |
| KU1 | C18693T | S218S | NSP14 | 0 | 0.00% | 790 | 64.44% | 0 | 0.00% | 436 | 35.56% | 0 | 0.00% |
| KU1 | G18697A | A220T | NSP14 | 489 | 38.44% | 0 | 0.00% | 783 | 61.56% | 0 | 0.00% | 0 | 0.00% |
| KU1 | A19592G | N518S | NSP14 | 32 | 35.56% | 0 | 0.00% | 57 | 63.33% | 1 | 1.11% | 0 | 0.00% |
| KU1 | A21493G | R279G | NSP16 | 411 | 69.78% | 0 | 0.00% | 178 | 30.22% | 0 | 0.00% | 0 | 0.00% |
| KU1 | C21618G | T19R | Spike | 0 | 0.00% | 417 | 53.95% | 356 | 46.05% | 0 | 0.00% | 0 | 0.00% |
| KU1 | T21819A | F86Y | Spike | 143 | 44.41% | 0 | 0.00% | 0 | 0.00% | 179 | 55.59% | 0 | 0.00% |
| KU1 | C21846T | T95I | Spike | 0 | 0.00% | 101 | 53.16% | 0 | 0.00% | 89 | 46.84% | 0 | 0.00% |
| KU1 | C22444T | D294D | Spike | 0 | 0.00% | 79 | 46.75% | 0 | 0.00% | 90 | 53.25% | 0 | 0.00% |
| KU1 | C22560T | T333I | Spike | 0 | 0.00% | 103 | 61.68% | 1 | 0.60% | 63 | 37.72% | 0 | 0.00% |
| KU1 | C22995A | T478K | Spike | 44 | 30.14% | 102 | 69.86% | 0 | 0.00% | 0 | 0.00% | 0 | 0.00% |
| KU1 | A23169G | N536S | Spike | 60 | 67.42% | 0 | 0.00% | 29 | 32.58% | 0 | 0.00% | 0 | 0.00% |
| KU1 | A23403G | D614G | Spike | 0 | 0.00% | 0 | 0.00% | 271 | 100.00% | 0 | 0.00% | 0 | 0.00% |
| KU1 | G24410A | D950N | Spike | 702 | 56.89% | 0 | 0.00% | 532 | 43.11% | 0 | 0.00% | 0 | 0.00% |
| KU1 | C25096T | N1178N | Spike | 0 | 0.00% | 5 | 29.41% | 0 | 0.00% | 12 | 70.59% | 0 | 0.00% |
| KU1 | C25463T | T24I | ORF3a | 0 | 0.00% | 313 | 69.56% | 0 | 0.00% | 137 | 30.44% | 0 | 0.00% |
| KU1 | G25563T | Q57H | ORF3a | 0 | 0.00% | 0 | 0.00% | 322 | 47.70% | 353 | 52.30% | 0 | 0.00% |
| KU1 | G25621A | V77I | ORF3a | 198 | 47.03% | 0 | 0.00% | 223 | 52.97% | 0 | 0.00% | 0 | 0.00% |
| KU1 | C26060T | T223I | ORF3a | 0 | 0.00% | 1 | 0.17% | 0 | 0.00% | 602 | 99.83% | 0 | 0.00% |
| KU1 | C26192T | P267L | ORF3a | 0 | 0.00% | 552 | 72.35% | 0 | 0.00% | 211 | 27.65% | 0 | 0.00% |
| KU1 | T26767C | I82T | Membrane | 0 | 0.00% | 1620 | 60.25% | 1 | 0.04% | 1068 | 39.72% | 0 | 0.00% |
| KU1 | A26864G | P114P | Membrane | 1501 | 58.47% | 3 | 0.12% | 1063 | 41.41% | 0 | 0.00% | 0 | 0.00% |
| KU1 | T28813C | S180S | Nucleocapsid | 6 | 0.11% | 1599 | 29.60% | 2 | 0.04% | 3795 | 70.25% | 0 | 0.00% |
| KU1 | G28881T | R203M | Nucleocapsid | 33 | 2.72% | 1 | 0.08% | 241 | 19.85% | 939 | 77.35% | 0 | 0.00% |
| KU1 | G29402T | D377Y | Nucleocapsid | 0 | 0.00% | 2 | 0.16% | 767 | 59.64% | 517 | 40.20% | 0 | 0.00% |
| KU1 | T29536C | 29536 | 3'UTR | 0 | 0.00% | 340 | 39.63% | 0 | 0.00% | 518 | 60.37% | 0 | 0.00% |
| SA2 | T1947C | V381A | NSP2 | 1 | 0.02% | 4509 | 99.78% | 1 | 0.02% | 8 | 0.18% | 0 | 0.00% |
| SA2 | G5567A | V950I | NSP3 | 96 | 48.73% | 0 | 0.00% | 101 | 51.27% | 0 | 0.00% | 0 | 0.00% |
| SA2 | A8040G | D1774G | NSP3 | 145 | 67.13% | 0 | 0.00% | 71 | 32.87% | 0 | 0.00% | 0 | 0.00% |
| SA2 | A8539G | I1940M | NSP3 | 619 | 33.92% | 4 | 0.22% | 1202 | 65.86% | 0 | 0.00% | 0 | 0.00% |
| SA2 | A8955G | Y134C | NSP4 | 55 | 58.51% | 0 | 0.00% | 39 | 41.49% | 0 | 0.00% | 0 | 0.00% |
| SA2 | C8986T | D144D | NSP4 | 0 | 0.00% | 66 | 34.92% | 0 | 0.00% | 123 | 65.08% | 0 | 0.00% |
| SA2 | G9053T | V167L | NSP4 | 0 | 0.00% | 2 | 0.58% | 93 | 26.88% | 251 | 72.54% | 0 | 0.00% |
| SA2 | C14408T | P314L | NSP12b | 0 | 0.00% | 2 | 0.28% | 0 | 0.00% | 709 | 99.72% | 0 | 0.00% |
| SA2 | T14482C | F339L | NSP12b | 1 | 0.08% | 589 | 46.16% | 0 | 0.00% | 686 | 53.76% | 0 | 0.00% |
| SA2 | A17205C | K323N | NSP13 | 4357 | 66.03% | 2233 | 33.84% | 9 | 0.14% | 0 | 0.00% | 0 | 0.00% |
| SA2 | G18716A | C226Y | NSP14 | 897 | 35.09% | 1 | 0.04% | 1651 | 64.59% | 7 | 0.27% | 0 | 0.00% |
| SA2 | C18877T | C279C | NSP14 | 3 | 0.22% | 22 | 1.60% | 1 | 0.07% | 1353 | 98.11% | 0 | 0.00% |
| SA2 | A20588G | D323G | NSP15 | 20 | 4.06% | 0 | 0.00% | 473 | 95.94% | 0 | 0.00% | 0 | 0.00% |
| SA2 | C22444T | D294D | Spike | 0 | 0.00% | 1 | 0.41% | 0 | 0.00% | 245 | 99.59% | 0 | 0.00% |
| SA2 | G22992A | S477N | Spike | 230 | 99.14% | 0 | 0.00% | 2 | 0.86% | 0 | 0.00% | 0 | 0.00% |
| SA2 | G23270T | A570S | Spike | 0 | 0.00% | 0 | 0.00% | 0 | 0.00% | 30 | 100.00% | 0 | 0.00% |
| SA2 | C23604G | P681R | Spike | 0 | 0.00% | 375 | 44.48% | 467 | 55.40% | 1 | 0.12% | 0 | 0.00% |
| SA2 | T23840C | C760R | Spike | 0 | 0.00% | 779 | 45.61% | 0 | 0.00% | 929 | 54.39% | 0 | 0.00% |
| SA2 | A23898T | Q779L | Spike | 291 | 52.43% | 3 | 0.54% | 0 | 0.00% | 261 | 47.03% | 0 | 0.00% |
| SA2 | A23942G | I794V | Spike | 83 | 59.71% | 0 | 0.00% | 56 | 40.29% | 0 | 0.00% | 0 | 0.00% |
| SA2 | G24368C | D936H | Spike | 0 | 0.00% | 958 | 100.00% | 0 | 0.00% | 0 | 0.00% | 0 | 0.00% |
| SA2 | T24514C | L984L | Spike | 0 | 0.00% | 385 | 30.58% | 0 | 0.00% | 874 | 69.42% | 0 | 0.00% |
| SA2 | A24647G | M1029V | Spike | 1513 | 69.50% | 1 | 0.05% | 663 | 30.45% | 0 | 0.00% | 0 | 0.00% |
| SA2 | G24781T | K1073N | Spike | 0 | 0.00% | 0 | 0.00% | 301 | 66.59% | 151 | 33.41% | 0 | 0.00% |
| SA2 | T25556G | V55G | ORF3a | 0 | 0.00% | 0 | 0.00% | 91 | 100.00% | 0 | 0.00% | 0 | 0.00% |
| SA2 | G25563T | Q57H | ORF3a | 0 | 0.00% | 0 | 0.00% | 0 | 0.00% | 97 | 100.00% | 0 | 0.00% |
| SA2 | C26735T | Y71Y | Membrane | 0 | 0.00% | 5 | 0.18% | 0 | 0.00% | 2733 | 99.82% | 0 | 0.00% |
| SA2 | A27606G | V71V | ORF7a | 89 | 74.79% | 0 | 0.00% | 30 | 25.21% | 0 | 0.00% | 0 | 0.00% |
| SA2 | T27638C | V82A | ORF7a | 0 | 0.00% | 23 | 23.96% | 0 | 0.00% | 73 | 76.04% | 0 | 0.00% |
| SA2 | C28854T | S194L | Nucleocapsid | 0 | 0.00% | 655 | 29.42% | 0 | 0.00% | 1571 | 70.58% | 0 | 0.00% |
| SA2 | DEL: 29441 | del_29441 | Nucleocapsid | 0 | 0.00% | 2 | 100.00% | 0 | 0.00% | 0 | 0.00% | 21 | 91.30% |
| SA4 | G529C | L88L | NSP1 | 0 | 0.00% | 219 | 23.80% | 701 | 76.20% | 0 | 0.00% | 0 | 0.00% |
| SA4 | A918G | Q38R | NSP2 | 223 | 71.02% | 0 | 0.00% | 91 | 28.98% | 0 | 0.00% | 0 | 0.00% |
| SA4 | C934T | D43D | NSP2 | 0 | 0.00% | 133 | 45.24% | 0 | 0.00% | 161 | 54.76% | 0 | 0.00% |
| SA4 | G984T | W60L | NSP2 | 0 | 0.00% | 2 | 0.14% | 496 | 35.84% | 886 | 64.02% | 0 | 0.00% |
| SA4 | T1947C | V381A | NSP2 | 0 | 0.00% | 1550 | 39.21% | 4 | 0.10% | 2399 | 60.69% | 0 | 0.00% |
| SA4 | T2575C | T590T | NSP2 | 0 | 0.00% | 3 | 30.00% | 0 | 0.00% | 7 | 70.00% | 0 | 0.00% |
| SA4 | C3037T | F106F | NSP3 | 0 | 0.00% | 0 | 0.00% | 0 | 0.00% | 13 | 100.00% | 0 | 0.00% |
| SA4 | A3069G | D117G | NSP3 | 6 | 46.15% | 0 | 0.00% | 7 | 53.85% | 0 | 0.00% | 0 | 0.00% |
| SA4 | G5015T | V766L | NSP3 | 0 | 0.00% | 1 | 0.30% | 1 | 0.30% | 330 | 99.40% | 0 | 0.00% |
| SA4 | C14408T | P314L | NSP12b | 0 | 0.00% | 0 | 0.00% | 0 | 0.00% | 60 | 100.00% | 0 | 0.00% |
| SA4 | T19209G | DR390EG | NSP14 | 0 | 0.00% | 0 | 0.00% | 28 | 93.33% | 2 | 6.67% | 0 | 0.00% |
| SA4 | A19210G |  |  | 2 | 6.67% | 0 | 0.00% | 28 | 93.33% | 0 | 0.00% | 0 | 0.00% |
| SA4 | A19212G | R391R | NSP14 | 2 | 6.67% | 0 | 0.00% | 28 | 93.33% | 0 | 0.00% | 0 | 0.00% |
| SA4 | A19214G | Y392C | NSP14 | 4 | 12.90% | 0 | 0.00% | 27 | 87.10% | 0 | 0.00% | 0 | 0.00% |
| SA4 | C19217A | P393H | NSP14 | 31 | 93.94% | 2 | 6.06% | 0 | 0.00% | 0 | 0.00% | 0 | 0.00% |
| SA4 | T21591C | L10P | Spike | 0 | 0.00% | 305 | 62.24% | 0 | 0.00% | 185 | 37.76% | 0 | 0.00% |
| SA4 | T22143A | F194Y | Spike | 54 | 28.13% | 0 | 0.00% | 0 | 0.00% | 138 | 71.88% | 0 | 0.00% |
| SA4 | C22236A | P225Q | Spike | 181 | 31.26% | 397 | 68.57% | 0 | 0.00% | 1 | 0.17% | 0 | 0.00% |
| SA4 | C22376T | P272S | Spike | 0 | 0.00% | 347 | 40.73% | 0 | 0.00% | 505 | 59.27% | 0 | 0.00% |
| SA4 | G23006T | G482C | Spike | 0 | 0.00% | 1 | 0.30% | 228 | 69.09% | 101 | 30.61% | 0 | 0.00% |
| SA4 | G23096T | V512L | Spike | 0 | 0.00% | 0 | 0.00% | 100 | 71.94% | 39 | 28.06% | 0 | 0.00% |
| SA4 | A23403G | D614G | Spike | 4 | 0.75% | 0 | 0.00% | 529 | 99.25% | 0 | 0.00% | 0 | 0.00% |
| SA4 | C23604G | P681R | Spike | 0 | 0.00% | 767 | 54.98% | 627 | 44.95% | 1 | 0.07% | 0 | 0.00% |
| SA4 | G25621A | V77I | ORF3a | 415 | 99.28% | 0 | 0.00% | 3 | 0.72% | 0 | 0.00% | 0 | 0.00% |
| SA4 | C26060T | T223I | ORF3a | 0 | 0.00% | 463 | 29.51% | 0 | 0.00% | 1106 | 70.49% | 0 | 0.00% |
| SA4 | C26735T | Y71Y | Membrane | 1 | 0.06% | 2 | 0.11% | 1 | 0.06% | 1813 | 99.78% | 0 | 0.00% |
| SA4 | A27947G | Q18Q | ORF8 | 191 | 71.00% | 0 | 0.00% | 78 | 29.00% | 0 | 0.00% | 0 | 0.00% |
| SA4 | G28881T | R203M | Nucleocapsid | 53 | 3.70% | 2 | 0.14% | 591 | 41.24% | 787 | 54.92% | 0 | 0.00% |
| SA6 | T18633C | F198F | NSP14 | 1 | 0.03% | 2311 | 72.58% | 0 | 0.00% | 872 | 27.39% | 0 | 0.00% |
| SA6 | C18877T | C279C | NSP14 | 2 | 0.06% | 1866 | 60.04% | 1 | 0.03% | 1239 | 39.86% | 0 | 0.00% |
| SA6 | T19209G | DR390EG | NSP14 | 0 | 0.00% | 0 | 0.00% | 30 | 100.00% | 0 | 0.00% | 0 | 0.00% |
| SA6 | A19210G |  |  | 0 | 0.00% | 0 | 0.00% | 30 | 100.00% | 0 | 0.00% | 0 | 0.00% |
| SA6 | A19212G | R391R | NSP14 | 0 | 0.00% | 0 | 0.00% | 30 | 100.00% | 0 | 0.00% | 0 | 0.00% |
| SA6 | A19214G | Y392C | NSP14 | 0 | 0.00% | 0 | 0.00% | 30 | 100.00% | 0 | 0.00% | 0 | 0.00% |
| SA6 | C19217A | P393H | NSP14 | 33 | 100.00% | 0 | 0.00% | 0 | 0.00% | 0 | 0.00% | 0 | 0.00% |
| SA6 | T21571C | V3V | Spike | 0 | 0.00% | 268 | 36.17% | 0 | 0.00% | 473 | 63.83% | 0 | 0.00% |
| SA6 | C21618G | T19R | Spike | 2 | 0.11% | 217 | 11.72% | 1632 | 88.17% | 0 | 0.00% | 0 | 0.00% |
| SA6 | C21635T | P25S | Spike | 3 | 0.13% | 222 | 9.53% | 0 | 0.00% | 2105 | 90.34% | 0 | 0.00% |
| SA6 | G21761T | A67S | Spike | 2 | 0.06% | 4 | 0.13% | 0 | 0.00% | 3174 | 99.81% | 0 | 0.00% |
| SA6 | C21846T | T95I | Spike | 0 | 0.00% | 0 | 0.00% | 1 | 0.06% | 1667 | 99.94% | 0 | 0.00% |
| SA6 | C21859T | N99N | Spike | 0 | 0.00% | 2 | 0.15% | 3 | 0.22% | 1366 | 99.64% | 0 | 0.00% |
| SA6 | C22444T | D294D | Spike | 0 | 0.00% | 0 | 0.00% | 0 | 0.00% | 144 | 100.00% | 0 | 0.00% |
| SA6 | A24652G | S1030S | Spike | 9 | 60.00% | 0 | 0.00% | 6 | 40.00% | 0 | 0.00% | 0 | 0.00% |
| SA6 | G25563T | Q57H | ORF3a | 0 | 0.00% | 4 | 0.68% | 0 | 0.00% | 582 | 99.32% | 0 | 0.00% |
| SA6 | C26060T | T223I | ORF3a | 0 | 0.00% | 2 | 0.69% | 0 | 0.00% | 286 | 99.31% | 0 | 0.00% |
| SA6 | C26735T | Y71Y | Membrane | 0 | 0.00% | 1 | 0.25% | 0 | 0.00% | 406 | 99.75% | 0 | 0.00% |
| SA6 | G28881A | RG203KR | Nucleocapsid | 261 | 93.88% | 0 | 0.00% | 15 | 5.40% | 2 | 0.72% | 0 | 0.00% |
| SA6 | G28882A |  |  | 261 | 98.12% | 0 | 0.00% | 5 | 1.88% | 0 | 0.00% | 0 | 0.00% |
| SA6 | G28883C |  |  | 0 | 0.00% | 266 | 100.00% | 0 | 0.00% | 0 | 0.00% | 0 | 0.00% |
| SA6 | DEL: 22216 | del_22216 | Spike | 5 | 62.50% | 0 | 0.00% | 3 | 37.50% | 0 | 0.00% | 363 | 97.80% |
| SA7 | G984T | W60L | NSP2 | 1 | 0.40% | 1 | 0.40% | 40 | 16.06% | 207 | 83.13% | 0 | 0.00% |
| SA7 | G11230T | M86I | NSP6 | 11 | 0.35% | 16 | 0.51% | 276 | 8.71% | 2864 | 90.43% | 0 | 0.00% |
| SA7 | C14408T | P314L | NSP12b | 0 | 0.00% | 1 | 0.68% | 0 | 0.00% | 145 | 99.32% | 0 | 0.00% |
| SA7 | C19547T | S503L | NSP14 | 0 | 0.00% | 102 | 70.83% | 0 | 0.00% | 42 | 29.17% | 0 | 0.00% |
| SA7 | T21525C | V289V | NSP16 | 0 | 0.00% | 53 | 28.80% | 0 | 0.00% | 131 | 71.20% | 0 | 0.00% |
| SA7 | C21646T | Y28Y | Spike | 0 | 0.00% | 36 | 11.15% | 0 | 0.00% | 287 | 88.85% | 0 | 0.00% |
| SA7 | A23403G | D614G | Spike | 0 | 0.00% | 0 | 0.00% | 366 | 100.00% | 0 | 0.00% | 0 | 0.00% |
| SA7 | G25621A | V77I | ORF3a | 84 | 84.85% | 0 | 0.00% | 15 | 15.15% | 0 | 0.00% | 0 | 0.00% |
| SA7 | C26060T | T223I | ORF3a | 0 | 0.00% | 44 | 4.49% | 0 | 0.00% | 936 | 95.51% | 0 | 0.00% |
| SA7 | A27947G | Q18Q | ORF8 | 94 | 20.66% | 0 | 0.00% | 361 | 79.34% | 0 | 0.00% | 0 | 0.00% |
| SA7 | G28881T | R203M | Nucleocapsid | 218 | 11.18% | 0 | 0.00% | 847 | 43.44% | 885 | 45.38% | 0 | 0.00% |
| SA7 | G28916T | G215C | Nucleocapsid | 0 | 0.00% | 0 | 0.00% | 4 | 26.67% | 11 | 73.33% | 0 | 0.00% |
| SA7 | C29149T | I292I | Nucleocapsid | 0 | 0.00% | 2 | 11.11% | 0 | 0.00% | 16 | 88.89% | 0 | 0.00% |
| SA7 | A29237T | M322L | Nucleocapsid | 158 | 61.00% | 0 | 0.00% | 5 | 1.93% | 96 | 37.07% | 0 | 0.00% |
| SA7 | A29357G | T362A | Nucleocapsid | 327 | 62.88% | 1 | 0.19% | 192 | 36.92% | 0 | 0.00% | 0 | 0.00% |
| SA7 | G29402T | D377Y | Nucleocapsid | 0 | 0.00% | 0 | 0.00% | 333 | 67.27% | 162 | 32.73% | 0 | 0.00% |
| SA9 | C745T | N160N | NSP1 | 0 | 0.00% | 0 | 0.00% | 0 | 0.00% | 241 | 100.00% | 0 | 0.00% |
| SA9 | A846G | D14G | NSP2 | 273 | 72.99% | 0 | 0.00% | 101 | 27.01% | 0 | 0.00% | 0 | 0.00% |
| SA9 | T2136C | L444P | NSP2 | 1 | 0.02% | 4640 | 99.87% | 0 | 0.00% | 5 | 0.11% | 0 | 0.00% |
| SA9 | T4150C | V477V | NSP3 | 0 | 0.00% | 83 | 100.00% | 0 | 0.00% | 0 | 0.00% | 0 | 0.00% |
| SA9 | G4181T | A488S | NSP3 | 0 | 0.00% | 0 | 0.00% | 0 | 0.00% | 90 | 100.00% | 0 | 0.00% |
| SA9 | C4327T | Y536Y | NSP3 | 0 | 0.00% | 0 | 0.00% | 0 | 0.00% | 10 | 100.00% | 0 | 0.00% |
| SA9 | C7124T | P1469S | NSP3 | 0 | 0.00% | 1 | 0.17% | 0 | 0.00% | 593 | 99.83% | 0 | 0.00% |
| SA9 | T7154C | S1479P | NSP3 | 1 | 0.11% | 673 | 71.52% | 0 | 0.00% | 267 | 28.37% | 0 | 0.00% |
| SA9 | A7180G | E1487E | NSP3 | 897 | 73.95% | 0 | 0.00% | 316 | 26.05% | 0 | 0.00% | 0 | 0.00% |
| SA9 | T7348C | N1543N | NSP3 | 0 | 0.00% | 260 | 29.48% | 0 | 0.00% | 622 | 70.52% | 0 | 0.00% |
| SA9 | T7378C | L1553L | NSP3 | 1 | 0.15% | 456 | 66.38% | 0 | 0.00% | 230 | 33.48% | 0 | 0.00% |
| SA9 | A11332G | V120V | NSP6 | 0 | 0.00% | 0 | 0.00% | 24 | 100.00% | 0 | 0.00% | 0 | 0.00% |
| SA9 | G11365T | V131V | NSP6 | 0 | 0.00% | 0 | 0.00% | 2 | 4.65% | 41 | 95.35% | 0 | 0.00% |
| SA9 | A11474G | I168V | NSP6 | 69 | 56.56% | 0 | 0.00% | 53 | 43.44% | 0 | 0.00% | 0 | 0.00% |
| SA9 | G12223A | V44V | NSP8 | 39 | 28.68% | 0 | 0.00% | 97 | 71.32% | 0 | 0.00% | 0 | 0.00% |
| SA9 | G12753A | C23Y | NSP9 | 1052 | 26.60% | 0 | 0.00% | 2902 | 73.38% | 1 | 0.03% | 0 | 0.00% |
| SA9 | G12767A | A28T | NSP9 | 1032 | 27.32% | 0 | 0.00% | 2744 | 72.65% | 1 | 0.03% | 0 | 0.00% |
| SA9 | T13216C | D64D | NSP10 | 0 | 0.00% | 573 | 34.60% | 0 | 0.00% | 1083 | 65.40% | 0 | 0.00% |
| SA9 | A13327G | T101T | NSP10 | 1827 | 74.15% | 1 | 0.04% | 635 | 25.77% | 1 | 0.04% | 0 | 0.00% |
| SA9 | T13402G | Y126* | NSP10 | 0 | 0.00% | 3 | 0.13% | 765 | 33.88% | 1490 | 65.99% | 0 | 0.00% |
| SA9 | C13432G | P136P | NSP10 | 2 | 0.12% | 1106 | 63.64% | 628 | 36.13% | 2 | 0.12% | 0 | 0.00% |
| SA9 | A13838G | H124R | NSP12b | 52 | 36.88% | 0 | 0.00% | 89 | 63.12% | 0 | 0.00% | 0 | 0.00% |
| SA9 | C14119G | P218A | NSP12b | 0 | 0.00% | 12 | 52.17% | 11 | 47.83% | 0 | 0.00% | 0 | 0.00% |
| SA9 | T14745C | V426V | NSP12b | 0 | 0.00% | 447 | 100.00% | 0 | 0.00% | 0 | 0.00% | 0 | 0.00% |
| SA9 | A14862G | E465E | NSP12b | 0 | 0.00% | 0 | 0.00% | 771 | 100.00% | 0 | 0.00% | 0 | 0.00% |
| SA9 | G16158A | M897I | NSP12b | 34 | 73.91% | 0 | 0.00% | 12 | 26.09% | 0 | 0.00% | 0 | 0.00% |
| SA9 | T16254A | V6V | NSP13 | 139 | 83.73% | 1 | 0.60% | 0 | 0.00% | 26 | 15.66% | 0 | 0.00% |
| SA9 | C16466T | P77L | NSP13 | 0 | 0.00% | 1 | 2.17% | 0 | 0.00% | 45 | 97.83% | 0 | 0.00% |
| SA9 | A17203G | K323E | NSP13 | 2526 | 32.06% | 0 | 0.00% | 5352 | 67.94% | 0 | 0.00% | 0 | 0.00% |
| SA9 | T17348C | V371A | NSP13 | 1 | 0.02% | 1797 | 40.48% | 2 | 0.05% | 2639 | 59.45% | 0 | 0.00% |
| SA9 | C18693T | S218S | NSP14 | 4 | 0.05% | 4845 | 61.81% | 1 | 0.01% | 2988 | 38.12% | 0 | 0.00% |
| SA9 | C19220T | A394V | NSP14 | 0 | 0.00% | 3 | 0.26% | 0 | 0.00% | 1155 | 99.74% | 0 | 0.00% |
| SA9 | A19945T | K109* | NSP15 | 911 | 57.40% | 3 | 0.19% | 0 | 0.00% | 673 | 42.41% | 0 | 0.00% |
| SA9 | C19955T | T112I | NSP15 | 0 | 0.00% | 4 | 0.25% | 1 | 0.06% | 1619 | 99.69% | 0 | 0.00% |
| SA9 | T20605C | F329L | NSP15 | 0 | 0.00% | 246 | 100.00% | 0 | 0.00% | 0 | 0.00% | 0 | 0.00% |
| SA9 | A21894T | D111V | Spike | 1 | 6.67% | 0 | 0.00% | 0 | 0.00% | 14 | 93.33% | 0 | 0.00% |
| SA9 | G21987A | G142D | Spike | 25 | 49.02% | 0 | 0.00% | 26 | 50.98% | 0 | 0.00% | 0 | 0.00% |
| SA9 | T22917G | L452R | Spike | 3 | 0.08% | 6 | 0.16% | 1861 | 50.20% | 1837 | 49.55% | 0 | 0.00% |
| SA9 | C22995A | T478K | Spike | 817 | 49.25% | 840 | 50.63% | 0 | 0.00% | 2 | 0.12% | 0 | 0.00% |
| SA9 | T24085A | L841L | Spike | 732 | 28.94% | 0 | 0.00% | 2 | 0.08% | 1795 | 70.98% | 0 | 0.00% |
| SA9 | T24151C | P863P | Spike | 3 | 0.14% | 569 | 27.45% | 1 | 0.05% | 1500 | 72.36% | 0 | 0.00% |
| SA9 | A25385G | 25385 | 3'UTR | 2 | 1.02% | 0 | 0.00% | 195 | 98.98% | 0 | 0.00% | 0 | 0.00% |
| SA9 | C25469T | S26L | ORF3a | 1 | 0.26% | 0 | 0.00% | 0 | 0.00% | 381 | 99.74% | 0 | 0.00% |
| SA9 | C26060T | T223I | ORF3a | 0 | 0.00% | 4 | 1.02% | 0 | 0.00% | 390 | 98.98% | 0 | 0.00% |
| SA9 | C26176T | P262S | ORF3a | 0 | 0.00% | 6 | 1.19% | 1 | 0.20% | 499 | 98.62% | 0 | 0.00% |
| SA9 | A26544G | I8V | Membrane | 33 | 53.23% | 0 | 0.00% | 29 | 46.77% | 0 | 0.00% | 0 | 0.00% |
| SA9 | A26564G | K14K | Membrane | 14 | 43.75% | 0 | 0.00% | 18 | 56.25% | 0 | 0.00% | 0 | 0.00% |
| SA9 | G26690T | L56L | Membrane | 1 | 0.02% | 10 | 0.24% | 2 | 0.05% | 4114 | 99.69% | 0 | 0.00% |
| SA9 | C27752T | T120I | ORF7a | 0 | 0.00% | 0 | 0.00% | 0 | 0.00% | 42 | 100.00% | 0 | 0.00% |
| SA9 | C27874T | T40I | ORF7b | 0 | 0.00% | 0 | 0.00% | 0 | 0.00% | 99 | 100.00% | 0 | 0.00% |
| SA9 | T28034C | I47I | ORF8 | 0 | 0.00% | 177 | 52.06% | 0 | 0.00% | 163 | 47.94% | 0 | 0.00% |
| SA9 | A28055T | S54S | ORF8 | 290 | 54.82% | 0 | 0.00% | 0 | 0.00% | 239 | 45.18% | 0 | 0.00% |
| SA9 | A28461G | D63G | Nucleocapsid | 0 | 0.00% | 0 | 0.00% | 1262 | 99.92% | 1 | 0.08% | 0 | 0.00% |
| SA9 | T28618C | T115T | Nucleocapsid | 2 | 0.03% | 1493 | 25.34% | 2 | 0.03% | 4394 | 74.59% | 0 | 0.00% |
| SA9 | A28778G | K169E | Nucleocapsid | 4419 | 73.75% | 0 | 0.00% | 1573 | 26.25% | 0 | 0.00% | 0 | 0.00% |
| SA9 | T28813C | S180S | Nucleocapsid | 0 | 0.00% | 1613 | 39.00% | 2 | 0.05% | 2521 | 60.95% | 0 | 0.00% |
| SA9 | C28831T | S186S | Nucleocapsid | 0 | 0.00% | 2225 | 64.47% | 0 | 0.00% | 1226 | 35.53% | 0 | 0.00% |
| SA9 | G28881T | R203M | Nucleocapsid | 5 | 0.35% | 9 | 0.62% | 2 | 0.14% | 1428 | 98.89% | 0 | 0.00% |
| SA9 | DEL: 28248-28253 | del_28248 | ORF8 | 78 | 44.32% | 0 | 0.00% | 95 | 53.98% | 3 | 1.70% | 975 | 84.70% |
| SA9 |  | del_28249 |  | 4 | 2.30% | 0 | 0.00% | 2 | 1.15% | 168 | 96.55% | 978 | 84.90% |
| SA9 |  | del_28250 |  | 0 | 0.00% | 72 | 43.11% | 0 | 0.00% | 95 | 56.89% | 977 | 85.40% |
| SA9 |  | del_28251 |  | 0 | 0.00% | 0 | 0.00% | 2 | 1.39% | 142 | 98.61% | 977 | 87.20% |
| SA9 |  | del_28252 |  | 44 | 31.65% | 0 | 0.00% | 0 | 0.00% | 95 | 68.35% | 979 | 87.60% |
| SA9 |  | del_28253 |  | 171 | 96.07% | 5 | 2.81% | 1 | 0.56% | 1 | 0.56% | 978 | 84.60% |


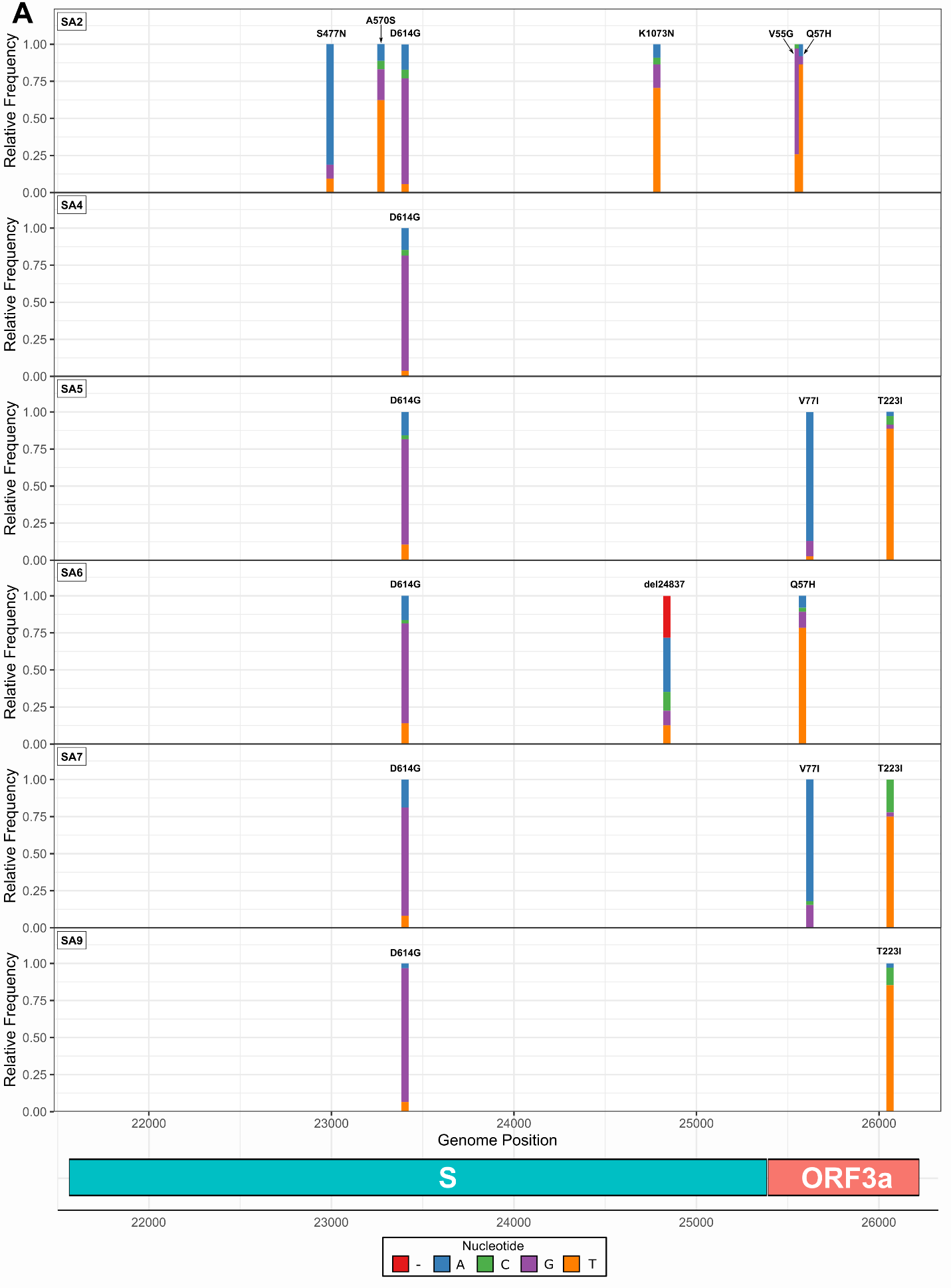

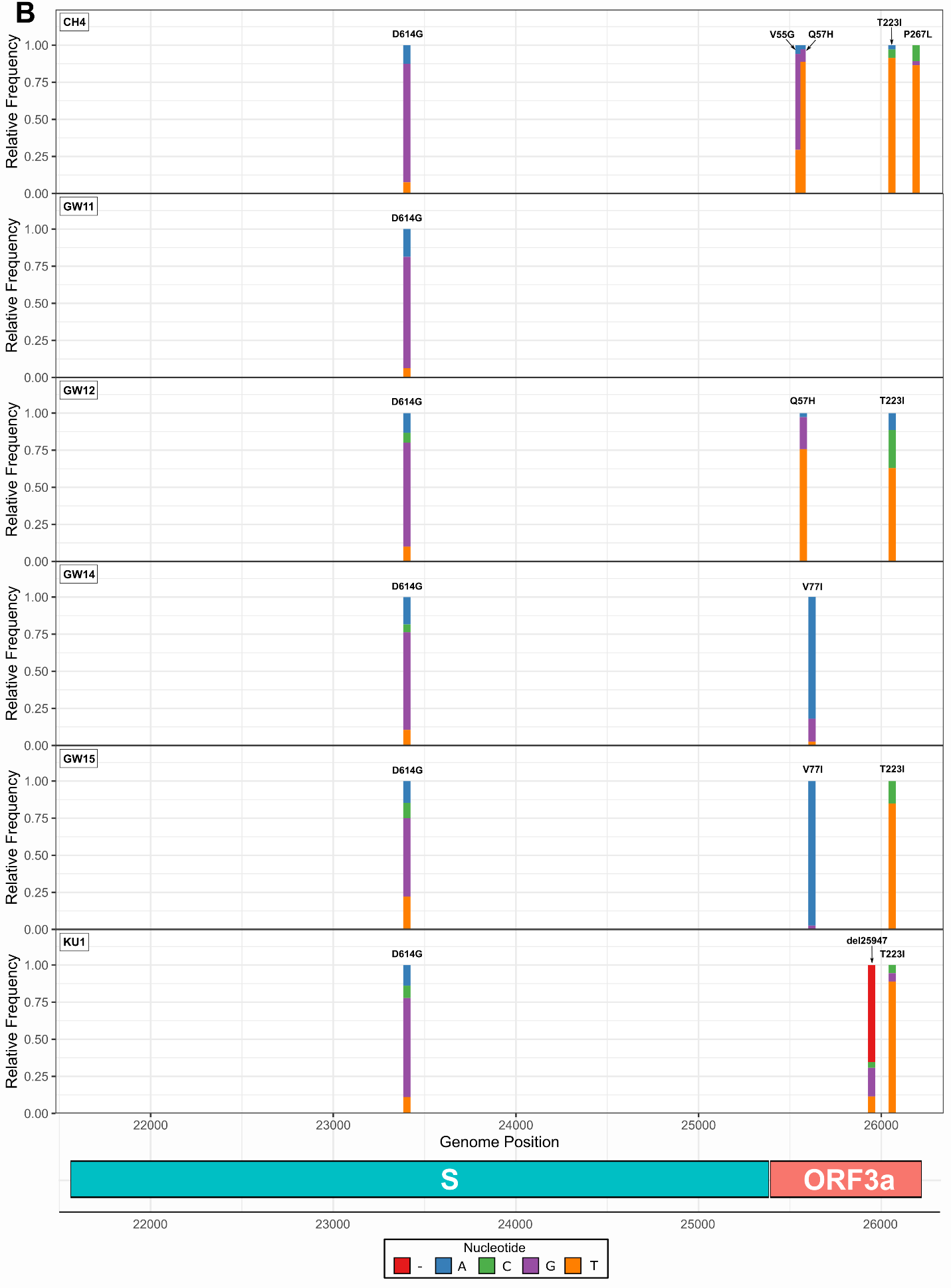

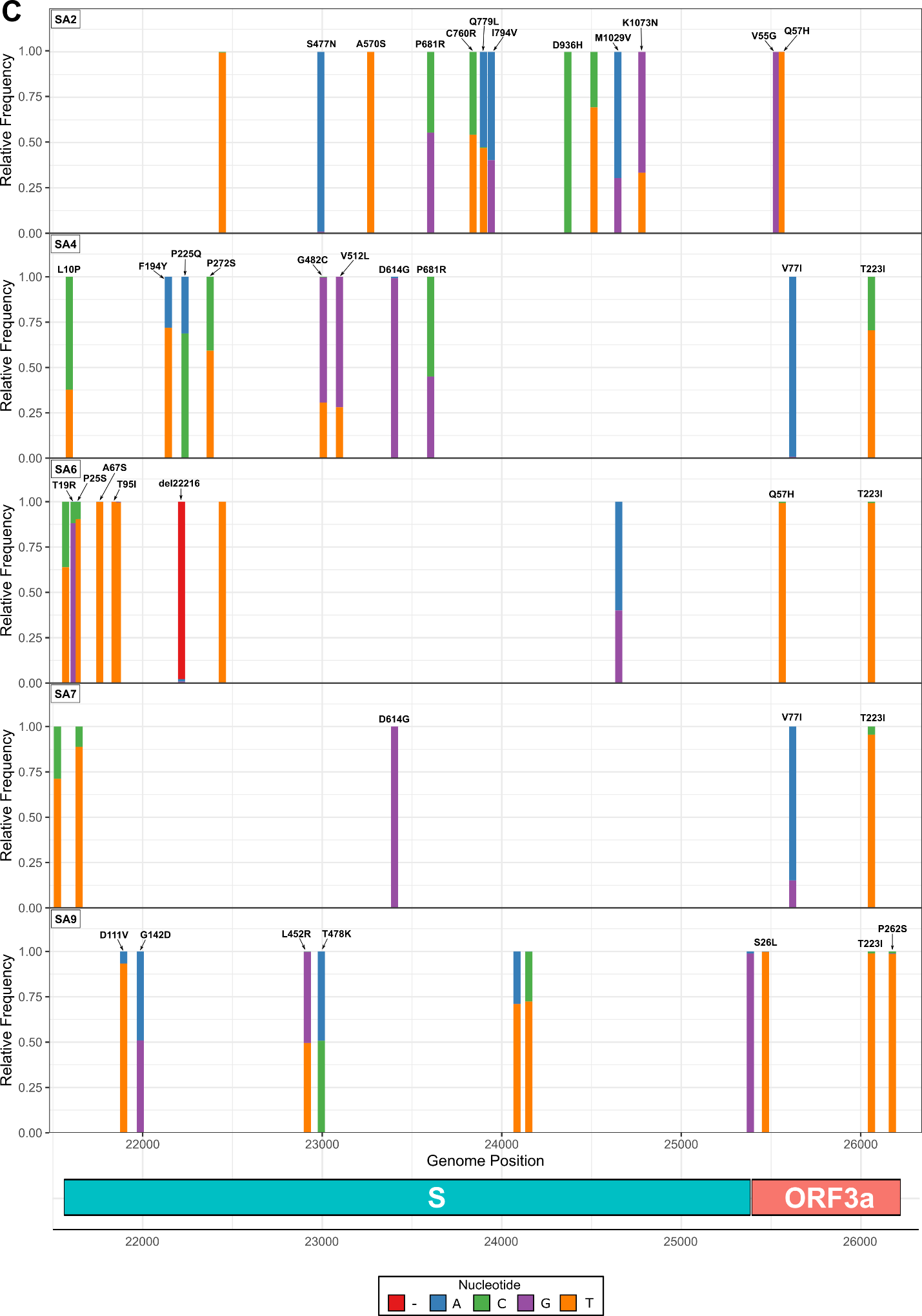

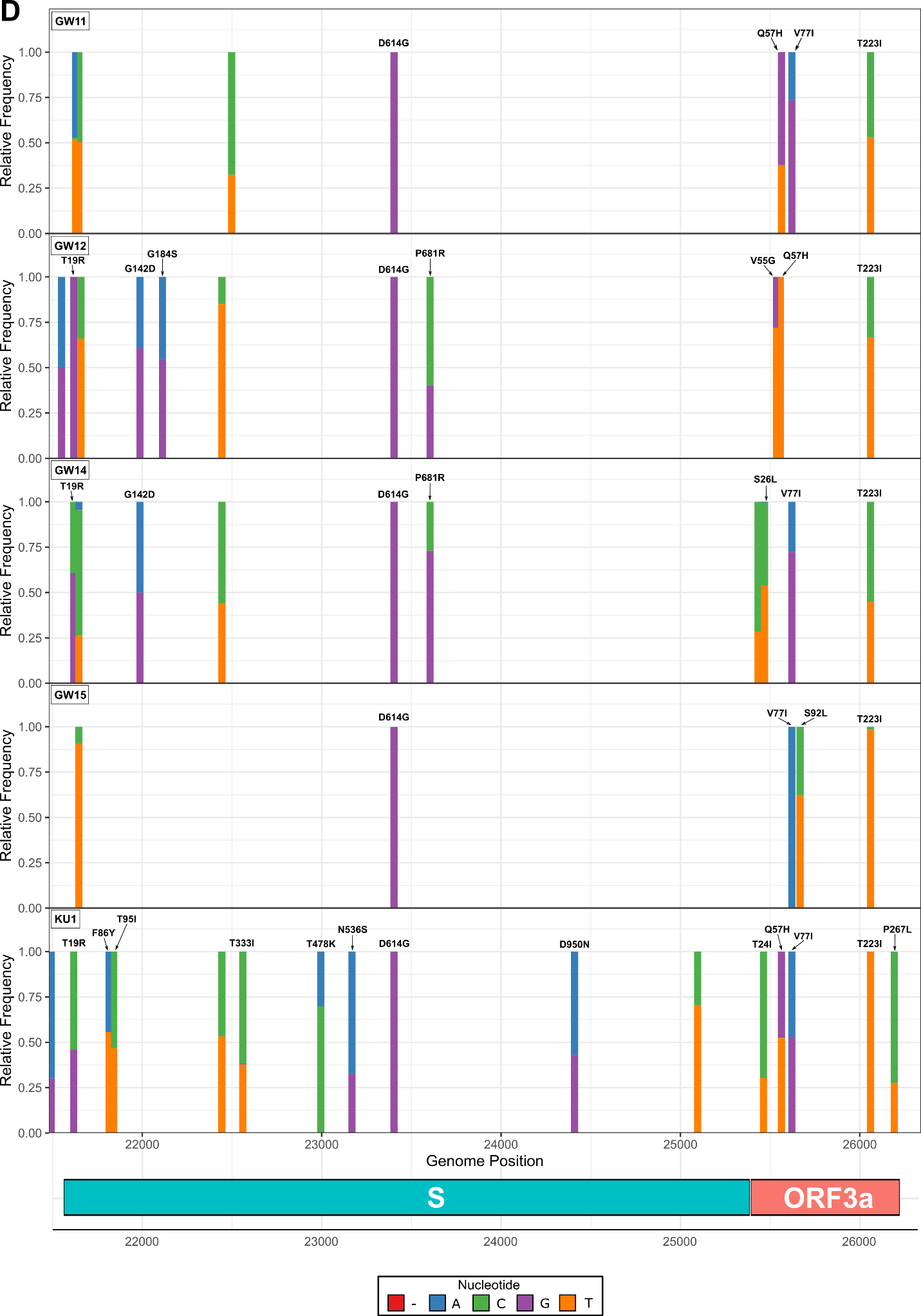


**Supplementary S1 Figure 1a and 1b Distribution of single nucleotide polymorphisms (SNP) and nucleotide deletions across the spike protein (S) and ORF3a genes of SARS-CoV-2 from the 12 sewage samples collected in this study**. (A, B) indicates nucleotide frequency of mutations on the S and ORF3a genes, generated during Nanopore MinION sequencing of the 12 wastewater samples collected in the study. (C, D) indicates nucleotide frequency of mutations on the S and ORF3a genes, generated during Illumina MiSeq sequencing of 10 of the wastewater samples. Nucleotide frequencies at each nucleotide position of the mutations are indicated with different colors (shown in key) to show nucleotide variation in the reads generated during Nanopore sequencing, obtained using Geneious Prime 2020.2.3. The S protein and ORF3a genes of the SARS-CoV-2 genome is schematically represented on the bottom of the plot. Amino acid mutations, SNPs and deletions have been annotated on the plot according to the presence of significant nucleotide frequency changes on each position, with the exclusion of labels for synonymous nucleotide mutations.
