# Supplementary material for: Rapid genomic surveillance of SARS-CoV-2 in a dense urban community of Kathmandu Valley using sewage samples
Source: PLoS One. 2023 Mar 30;18(3):e0283664. doi: 10.1371/journal.pone.0283664 (PMC10062583; doi:10.1371/journal.pone.0283664)
Supplement: Attachment — Submitted filename: ES Paper Reviewers comments.docx [file pone.0283664.s002.docx]

1. Is the manuscript technically sound, and do the data support the conclusions?

The manuscript must describe a technically sound piece of scientific research with data that supports the conclusions. Experiments must have been conducted rigorously, with appropriate controls, replication, and sample sizes. The conclusions must be drawn appropriately based on the data presented.

Reviewer #1: Partly

Reviewer #2: Yes

Reviewer #3: Partly

Reviewer #4: No

2. Has the statistical analysis been performed appropriately and rigorously?

Reviewer #1: Yes

Reviewer #2: Yes

Reviewer #3: N/A

Reviewer #4: Yes

3. Have the authors made all data underlying the findings in their manuscript fully available?

The [PLOS Data policy](http://www.plosone.org/static/policies.action#sharing) requires authors to make all data underlying the findings described in their manuscript fully available without restriction, with rare exception (please refer to the Data Availability Statement in the manuscript PDF file). The data should be provided as part of the manuscript or its supporting information, or deposited to a public repository. For example, in addition to summary statistics, the data points behind means, medians and variance measures should be available. If there are restrictions on publicly sharing data—e.g. participant privacy or use of data from a third party—those must be specified.

Reviewer #1: No

Reviewer #2: Yes

Reviewer #3: Yes

Reviewer #4: Yes

4. Is the manuscript presented in an intelligible fashion and written in standard English?

PLOS ONE does not copyedit accepted manuscripts, so the language in submitted articles must be clear, correct, and unambiguous. Any typographical or grammatical errors should be corrected at revision, so please note any specific errors here.

Reviewer #1: Yes

Reviewer #2: Yes

Reviewer #3: Yes

Reviewer #4: Yes

5. Review Comments to the Author

Reviewer #1:

Napit et al. present a manuscript detailing genomic surveillance of SARS-CoV-2 in sewage in the Kathmandu Valley. The authors demonstrate a technical approach for detailing SARS-CoV-2 genomic variation in sewage. The manuscript is well written and a valuable contribution to the growing body of literature on sewage surveillance for SARS-CoV-2. I have some concerns on the current data interpretation/presentation noted below for the authors’ consideration.

1. My primary technical concern is the inability of the genomic approach utilized to assemble complete genomes. Essentially, the sewage samples may include many different variants, and these variants may themselves be different than previously published variants. I still believe the study is valuable, but the presentation should be refined to show this uncertainty.

***As per the recommendation, variant analysis was conducted and has been included in the manuscript - line 219.***

2. With above – how were variants defined in Figure 6? Varinat was determined using Cov glue mentioned in line 314 to line 317

***Figure 6 has been removed. Cov Glue was still used but the data obtained from it was validated by identifying the variants using Geneious (VCF File analysis) – line 266-297***

3. Figure 5 – how was this heatmap constructed? Was qPCR or a similar approach used?

***Heat map has been reconstructed by normalizing incidence rate (using mean) in specific catchment areas – line 122-150***

4. Sequence data should be publicly deposited.

***The data has been publicly deposited on NCBI and GitHub in Data availability section- line 386.***

Reviewer #2:

The authors developed a genomic-based environmental surveillance system by detecting and characterizing SARS-CoV-2 in sewage samples of Kathmandu using portable next-generation DNA sequencing devices. This genomic-based environmental surveillance could help in the early detection of outbreaks and could provide unbiased information on a population level and detect shedding by symptomatic and asymptomatic patients. The latter is considered as the bulk of transmission. Moreover, this study could emphasize the importance of genomic surveillance as a key method of understanding viral evolution.

The manuscript overall is well presented. The method to comprehensively map sewage lines and to conduct field surveys to map out population size based on catchment area are well designed. Data analysis is done well. The idea behind the work holds relevance and is important to public health.

Reviewer #3:

SARS-CoV-2 monitoring in wastewater enables continuous surveillance of the incidence in a distinct region and can help to detect new hotspots before clinical case numbers increase. In addition, sequencing methods can be used to monitor the current SARS-CoV-2 mutations and their fixation in the population. In this manuscript, Napit et al. established a SARS-CoV-2 genomic surveillance workflow to monitor and characterize viral strains in the resource-limited country Nepal. NGS-sequencing (MiSeq) was compared to portable MinION sequencing.
The authors have shown that the MinION based method (ARTIC protocol, already well-described) has achieved comparable results to MiSeq sequencing, which however was already shown by others. I fully agree with the authors that in resource-limited regions like Nepal SARS-CoV-2 monitoring and sequencing is of enormous importance. Thus, it is very gratifying to see that sequencing could be established in the setting. However, the novelty for the readership of PLOS ONE is very limited.
A limitation of the manuscript is still that only a single sampling during the pandemic was shown, whereas surveillance implies continuous long-term monitoring. It would be desirable to see an decrease / increase in case numbers or mutations over time. The authors describe numerous tansitions and transversions were found, but this does not allow conclusions to be drawn about the mutation frequency. It is not possible to distinguish whether these are already introduced variants.

***Transitions and transversions have been removed as variant analysis were conducted – line 219***

Further comments:
The results partly read like a material and methods section and are repetitive. Furthermore, the reader lacks introductory sentences about what the purpose of this study was. Instead, there is a direct description of the methods and method comparisons. It would also be helpful to mention why the sampling sites were chosen for the study.

What exactly is the message of the mutations shown in Figure 4 and 7 (Waterfall plot)? This should be elaborated more clearly in the body text.

***The waterfall plot has been removed and replaced with heat map of comparative data (miseq and minion) -line 300/ figure 6 These are the mutations (with greater than 50% abundance in vcf file).***

***We had extensive data from the selected sampling sites from another project, which helped us to identify sewage catchment areas, population size and specific sampling sites (open sewage). Also, was received permission from the local government to conduct study on those sites.***

Is there any clinical data available, e.g. documented symptomatic cases? If so, it would be interesting to see a comparison to the sequencing data.

The figure legends should be revised since they are somehow repetitive and are also part of the body text.

***The government had only collected district level data during the time of sampling. We have added a calculated prevalence by normalizing the population data – figure 5 and supplementary data S6 table***

Reviewer #4:

Summary

The authors present a description of SARS-CoV-2 PCR and WGS analyses from a sample of waste water specimens for the purposes of pandemic surveillance. While this effort is potentially promising, it is difficult to draw any major conclusions from the data and analyses reported in this study. The major issues are that the sample sizes are very small, genome coverage is poor and there is no community incidence data to correlate findings with actual numbers of cases. It would be difficult to make any public health decisions with this data as it is now, particularly around early warning systems and relaxation of population-level non-pharmacologic interventions. Specific comments below.

Methods

1. Line 120: It would be important to show in the supplement the data supporting the equivalence of spot sampling and 24hr sampling

***24hr sampling had been added in supplementary data S4 Table.***

2. It is unclear what samples underwent RT-PCR, nested PCR and WGS and nowhere is it listed in the text how many total samples were analyzed.—

***Has been addressed in methodology section and table 1 has been added having that information. Line 91 to 112***

3. Line 143: The rationale for assessing the presence of non-SARS-CoV-2 coronaviruses is not clear as these are likely already endemic.--

***Has been added in line 80 to 89***
Results

1. Lines 251-255: It is not clear from the first results paragraph what the results of the optimization study actually are and how they pertain to the overall objective of the study. As such, the authors should consider moving this into the methods section or adding to the supplement. Moved this to methods section…..

***Completed***

2. Lines 253-255: Unclear how detection of other CoVs is relevant for study objectives. —

***Cov Glue was still used but the data obtained from it was validated by identifying the variants using Geneious (VCF File analysis) – line 219***

3. Lines 256-264: Comparison of MinION to MiSeq sequences: With a sample size of 2, it is hard to draw conclusions from this data. Further detail of which parts of the genome had the best coverage might also be helpful. The significance of the differences as reported in the manuscript are not known.—

***There are lot of literature available for MiSeq and MinION comparison and all samples has been re-sequenced with Miseq as well – line 198 and figure 6, supplementary data S9 Table.***

4. Table 1: I do not understand how to interpret this table. What do 'First', 'Second' and 'Third' run refer to? These are not mentioned in the Methods. Where are these samples coming from? Where does Table 1 refer to the MiSeq data (line 264)?—

***Old Table 1 has been removed.***

5. References could be mentioned on first use only (example: line 234 and line 267). Also GenVisR is inconsistently cited, sometimes as references 55 (line 325) and sometimes as reference 56 (line 328).—

***This has been fixed.***

6. Lines 285-290: The great deal of heterogeneity in the levels of RNA make it challenging to know how to interpret the data without accompanying incidence data of cases in the community. Other environmental surveillance efforts also normalize to background RNA from a difference virus.—

***We have calculated incidence rate by normalizing the population data using multiple literature - line 122 to 149***

7. Lines 297-304: For the purposes of surveillance for variants, the region of the genome containing the spike gene is of highest importance given its implications for natural infection or vaccine-induced protection and immune escape. It would be important for readers wishing to understand the utility of wastewater surveillance to know how well the spike gene was covered in this set of sequenced samples included in the final results.—

***Pangolin lineage analysis is only performed on consensus sequence, for sample variant analysis, overall SNPs have been analyzed along with spike gene – line 269***

8. Lines 319-320: This statement is unclear. Are the authors claiming the viruses circulating in their municipality are evolving at a rate faster than SARS-CoV-2 viruses elsewhere across the world? A comparison to the transition / transversion ratios seen in the single citation listed would also be helpful to place the results in context.—

***This has been removed.***

9. Lines 330-335: It is difficult to interpret the summary of mutations because genome coverage from the sewage samples is poor. It is therefore unknown what other mutations may be present in circulating strains have been missed due to their absence in the sequenced amplicons. These other mutations that make up the haplotype are critical to defining lineages, as shown in B.1.1.7.—

***SNP analysis has been conducted and percentage has been included in the graph. Also, pertinent table has been added in the supplement section. Supplementary data S6 to S9 table***

Discussion

1. Lines 362-365: Unclear how the detection of endemic CoVs is relevant for SARS-CoV-2 environmental surveillance.

***This has been removed.***

2. Lines 370: "the results obtained were almost identical" This does not seem supported by the data as the MiSeq data seemed quite different than the MinION data based on what was reported in the results.---

***MiSeq and MinION comparisons have been further elaborated in method, result and supplementary data S3 to S9 -line 185, 198, 270-297 and figure 6***

3. Lines 379-381: While it is true that the detection of other CoVs highlights the risk of emergence for a new CoV, the design of this study does not allow for drawing that conclusion because it is likely the case that other CoVs have always been present in the environment. If pre-pandemic surveillance had been able to show these CoVs are new, then that would support the statement.

***This has been removed.***

4. Lines 385-386: "Further studies can be designed to look into incidence estimation in communities and correlate that with available clinical data". This in fact is the key study. Without validation of SARS-CoV-2 RNA levels from sewage with community incidence data, it is not clear how environmental sampling can be used for informing public health interventions

***We have calculated incidence rate by normalizing the population data using multiple literature. Further, we are presenting this as a simple tool to estimate and indication for prevalence of SARS-CoV-2 with only simple viral load data- line 122-150 and 244-259.***

5. Lines 413-415: "Such a scenario creates an environment for the emergence of new variants, which was also the reason behind the origins of emerging variants in the UK (B.1.1.7), South Africa (B.1.351), and South America (P.1)" It is difficult to make this conclusion with the data presented. The variants of concern contain a large number of mutations that suddenly appeared (B.1.1.7 in particular), suggesting rapid evolution in an immunocompromised host. This is backed up by WGS from individual patients performed serially over time. It is not possible to say whether that is happening from environmental surveillance data, which by definition, integrates over many people’s virus lineages and lacks adequate genome coverage

***This has been removed.***

Figures

1. Fig 3: More appropriate for a supplement - ***removed***
2. Fig 4: Left hand panel X-axis is illegible and blue / red colors are not defined. – ***replaced by another figure***
3. Fig 5: 'Viral load intensity' is not defined – ***Normalized data has been used***
4. Fig 6: Colored dots are not defined. Is that a single sample? Multiple samples? This may be better represented with a table. – ***removed and replaced with table***
5. Fig 7: Same as Fig 4 - ***removed***
6. Fig 8: Definition of 'frequency' on the Y-axis is unclear. -***removed***
7. Fig 9: This figure is difficult to interpret. What do the colors on the large number of listed mutations signify? There is no way to know where a mutation lies on the genome using this color scheme – ***removed and replaced by another, easier to interpret figure in manuscript and supplementary data***

6. PLOS authors have the option to publish the peer review history of their article ([what does this mean?](https://journals.plos.org/plosone/s/editorial-and-peer-review-process#loc-peer-review-history)). If published, this will include your full peer review and any attached files.


If you choose “no”, your identity will remain anonymous but your review may still be made public.


**Do you want your identity to be public for this peer review?** For information about this choice, including consent withdrawal, please see our [Privacy Policy](https://www.plos.org/privacy-policy).

Reviewer #1: No

Reviewer #2: No

Reviewer #3: No

Reviewer #4: No

[NOTE: If reviewer comments were submitted as an attachment file, they will be attached to this email and accessible via the submission site. Please log into your account, locate the manuscript record, and check for the action link "View Attachments". If this link does not appear, there are no attachment files.]

- - - - -
For journal use only: PONEDEC3
