# Supplementary material for: Rapid genomic surveillance of SARS-CoV-2 in a dense urban community of Kathmandu Valley using sewage samples
Source: PLoS One. 2023 Mar 30;18(3):e0283664. doi: 10.1371/journal.pone.0283664 (PMC10062583; doi:10.1371/journal.pone.0283664)
Supplement: Attachment — Submitted filename: rebutal_letter.docx [file pone.0283664.s003.docx]

**CENTER FOR MOLECULAR DYNAMICS NEPAL**

[**www.cmdn.org**](http://www.cmdn.org)

**( PAN 302834428 SWC 24292)**

November 13, 2022

To,

The Editor

PLOS ONE

RE: Submission of our study “**Rapid genomic surveillance of SARS-CoV-2 in a dense urban community using**

**environmental (sewage) samples**”.

Dear Sir/Madam,

Greetings from the Center for Molecular Dynamics Nepal!

We are re-submitting our manuscript titled- “*Rapid genomic surveillance of SARS-CoV-2 in a dense urban community using environmental (sewage) samples*” with all the edits and changes as per reviewer’s comment.

We have addressed all the comments from editor, figure 1-3 has been double checked and placed in methodology section. Figure 1 and figure 5 caption has been updated to mention the copy right link and citation as per PLOS ONE requirement (<https://journals.plos.org/plosone/s/licenses-and-copyright>).

Further, financial disclosure we would like to be replaced with- “University of California-Davis and the USAID-funded PREDICT project for providing us with laboratory resources. Whole-genome sequencing was done at the Intrepid Nepal Genomic Center. Some of our work was partially funded by the Australian Development Agency and PSI grant (the Netherlands). Composite sampling robots were provided by Professor Eric Alm and Dr. Noriko Endo of BIOBOT (USA)”.

All these changes and additions are marked with “track changes” in its newer format. Along with this cover letter,

please find response to review letter responding to reviewers’ comments and suggestions. We believe these changes

substantially improved the manuscript and made it publication worthy.

We would like to thank the reviewers for their valuable feedback.

Sincerely,

Dibesh Karmacharya, Ph.D

Chairman/Executive Director

CMDN

5^th^ Floor Swaraj Sadan, Prasuti Griha Marg, Thapathali-11, Kathmandu

Ph +977 1 4251590; email: [info@cmdn.org](mailto:info@cmdn.org)

| Reviewer 1 | In the present manuscript PONE-D-22-13706 "Rapid genomic surveillance of SARS-CoV-2 in a dense urban community using environmental (sewage) samples", the authors have studied/presented the genomic surveillance of SARS-CoV-2 in wastewater samples Kathmandu, Nepal, by two different procedures, MiSeq (Illumina) and MinION (ONT). This is a well-written manuscript, the scope of which is really important in the current pandemic, and more important in countries like Nepal, as stated by the authors. | | |
| --- | --- | --- | --- |
|  | SNo | Comment | Response |
|  | 1 | The results of the study are not well presented, not really validated and moreover not novel for the readers. | The result section has been updated with more elaborate figure from supplementary data (figure 6) and table no. 3 has been added to make it more effective. |
|  | 2 | In this regard, the fig. 1-3 does not include any results but describing the sample sites/plan and the basic analytical procedures. | These figures are intended to elaborate the procedures/methodology and not the results- they are in methodology section. |
|  | 3 | Fig. 6, including the mutations/SNPs detected is poor, given the fact that WGS was performed. | The figure has been removed with the supplementary figure S1 which is more elaborate and depicts all the SNP with its relative abundance in y-axis while SNP position in x-axis. |

|  | 4 | The authors must present the % percentage of the mutations/SNPs detected and moreover to translate them to specific SARS-CoV-2 variants. | The mutation % has been added in supplementary table S3 and S9, Major circulating variant has been added in line 266-269 and table 3. The result is more specifically highlighted in the result section as well (line 301-304, 359-364 and 296-300). At the time of this study, variant of concerns was not classified and tools like Frejya that uses similar SNPs frequency-based algorithm and database containing all variant of concerns can actually predict circulation variant fractions (<https://github.com/andersen-lab/Freyja>) was not available. |
| --- | --- | --- | --- |
|  | 5 | Several discrepancies between MiSeq and MinION are presented. This is very important, and the authors should discuss it in detail. | The discrepancies has been highlighted both in result section line 292-293 Supplementary table S7. |
|  | 6 | The authors should describe in detail the quality control of the NGS libraries and the final sequencing output | The quality control has been added in line 190-193 and 202-206. |
|  | 7 | The quantification of viral load in WW samples and the "persons infected" formula are not clearly presented and described. | The formula was taken from the previous similar research paper; all the procedure was already described in those literature so was not mentioned earlier. The explanation has been added in line 135-137. |

|  | 8 | And most importantly, I cannot draw any specific and major conclusions from the results and discussion presented. | The discussion has been updated to add some clarity, we intended to demonstrate and highlight importance of genomic surveillance for disease like SARS-CoV-2. However, it is not feasible always for country like Nepal where fundings are not readily available, in such condition using low-cost sequencing to assess the situation and then if something important comes up then go for Miseq or clinical genomic surveillance of the community would be better option. Described in line 354-370, 380-392. |
| --- | --- | --- | --- |

| Reviewer 2 | Napit et al. studied the genomic surveillance of SARS-CoV-2 in sewage from Kathmandu, Nepal. The authors aimed to establish a genomic-based environmental surveillance system by monitoring SARS-CoV-2 in sewage samples via PCR and WGS approaches. Although the experiments in the manuscript are well-organized and the manuscript itself is also written in a good way, this reviewer can see right away the shortages of this study. The following are listed some major issues for the authors' consideration. | | |
| --- | --- | --- | --- |
|  | SNo | Comment | Response |
|  | 1 | One of the main issues is the data quality of the sequencing. I looked into some of the raw reads the authors deposited on GitHub. Since those data are directly related to the conclusions, it will not stand if such bad quality data was used. The author should consider toning down some of the conclusions from Fig. 6. | All the data was not of bad quality, few had low sequencing coverage, which is common in environmental samples as they do not have high viral load to that of clinical samples that has been acknowledged with all the table on coverage and depths in supplementary table and also in the manuscripts. The figure 6 has also been replaced with the Supplementary figure S1. Those low coverage samples had failed to distinguish lineage to high resolution (table no 3 has been added) for clarity. A line has been added in discussion highlighting the same (line 370-371) |
|  | 2 | Considering the environmental variants which might cause damage to lots of biological samples, the mutations the authors found has simply after the virus particles expose to sewage. It is essential to show some correlation to some clinical results to see if the mutations were presented in society. | The comparison with clinical samples were in discussion section but result was updated to contain variant calling data from consensus sequences in result section and table no. 3. |

|  | 3 | This reviewer really appreciates this kind of excellent effort in studying SARS-CoV-2 in developing countries like Nepal. The authors can discuss the significance of this study extensively since the novelty of this study is not apparent, especially in the methodology and results. One possible direction is to mention that sewage might become one of the possible infection routes for SARS-CoV-2, and we should be cautious since some mutations were found in environmental samples, such as sewage, in this study. | The discussion (line 385-390 and 392-395) has been updated to highlight the effort needed to track pandemic in country like Nepal cheaply and quickly in time. |
| --- | --- | --- | --- |
